# Cantrell Syndrome and the One Health Perspective: A Unified Review of Human and Comparative Cases

**DOI:** 10.3390/vetsci13020165

**Published:** 2026-02-07

**Authors:** Nieves Martín-Alguacil, Luis Avedillo

**Affiliations:** Research Group GIMCAD 971005-UCM, Departmental Section of Anatomy and Embryology, School of Veterinary Medicine, Universidad Complutense de Madrid, 28040 Madrid, Spain; luiavedi@ucm.es

**Keywords:** body stalk anomaly, Cantrell syndrome, Cardiac Embryology, comparative embryology, ectopia cordis, mesodermal development, one health, ventral body wall defects

## Abstract

Cantrell syndrome (CS) is a rare congenital disorder involving defects in the thoraco-abdominal midline, the diaphragm, the pericardium, the sternum and the heart. Since the initial description of the syndrome, 165 well-documented cases in humans have been reported, demonstrating substantial heterogeneity ranging from complete pentalogy to partial or atypical variants. A systematic review classified body wall defects and associated anomalies into nine categories, which are fully described in the manuscript. The categories include midline defects (UThAb, SUThAb, UAb, SUAb, SUICD, and UH), lateral defects (ThLAb and StLAb), and special cases. Each case was reassessed for umbilical cord status, body wall morphology, cardiac anomalies and additional malformations. Midline defects predominated (153 out of 165 cases, 92.7%), with supraumbilical variants being the most frequent. Umbilical hernias formed a distinct subgroup of ten cases. Lateral defects were uncommon (9 cases, 5.5%) and typically presented as thoracogastroschisis or lateral thoracoabdominoschisis. These defects were often associated with normal umbilical cords. Cardiac anomalies were universal, with ventricular and atrial septal defects being the most common findings. Reclassification revealed that many cases originally labeled as ‘classic pentalogy of Cantrell’ were more accurately classified as partial or atypical forms. This unified framework improves epidemiological understanding and diagnostic precision. From a One Health perspective, it underscores CS as a shared developmental vulnerability across mammalian species.

## Simple Summary

Cantrell syndrome (CS) is a rare condition affecting the development of the chest and abdominal wall, diaphragm, pericardium, sternum, and heart. Since the syndrome was first described in 1958, only 165 well-documented human cases have been reported, and they demonstrate a wide range of presentations. Some individuals have all five characteristic defects, while others exhibit partial or atypical combinations. Most cases involve midline defects above the umbilicus, though a few present with lateral openings or atypical patterns. Heart defects were present in every case, most often involving openings in the heart’s septa. Our review shows that many cases previously thought to represent the full “pentalogy” are better understood as partial or atypical forms. This study places CS within a broader developmental context by carefully examining the anatomy of the body wall and umbilical cord and comparing human findings with similar conditions in animals. The similarities between species highlight shared biological vulnerabilities and support a one health approach to studying congenital malformations.

## 1. Introduction

First described in 1958, Cantrell’s syndrome (CS) is a rare congenital disorder involving the disruption of multiple midline structures, including the sternum, diaphragm, pericardium, thoracic and abdominal walls, and heart [1]. This study reports 165 cases of Cantrell’s syndrome in human medicine [2,3,4,5,6,7,8,9,10,11,12,13,14,15,16,17,18,19,20,21,22,23,24,25,26,27,28,29,30,31,32,33,34,35,36,37,38,39,40,41,42,43,44,45,46,47,48,49,50,51,52,53,54,55,56,57,58,59,60,61,62,63,64,65,66,67,68,69,70,71,72,73,74,75,76,77,78,79,80,81,82,83,84,85,86,87,88,89,90,91,92,93,94,95,96,97,98,99,100,101,102,103]. However, the variability and inconsistent classification of these cases have hindered progress in understanding the developmental origins of the syndrome. The defining feature, *ectopia cordis* (EC), has been categorized in various ways, often conflating different pathological mechanisms and obscuring the embryological basis of these malformations [104]. From a developmental perspective, CS offers a valuable opportunity to study the fundamental processes of embryogenesis. During gastrulation, mesodermal cells migrate and differentiate into distinct lineages, including the lateral plate mesoderm [105,106,107,108]. This lineage contributes to the formation of the ventral body wall, diaphragm, pericardium, and cardiac structures. Proper fusion of the lateral plate mesoderm at the ventral midline is essential for continuity across thoracic and abdominal structures [105,106]. Failures in this fusion event can result in multisystem anomalies, including sternal clefts, diaphragmatic defects, and EC [105,106]. Conversely, lateral defects, such as gastroschisis, arise from localized disruptions in body wall folding. These defects reflect paraumbilical failures of morphogenetic closure, rather than systemic errors in mesodermal fusion [109,110,111,112,113,114,115]. It is important to recognize this distinction: midline defects represent global failures of embryonic integration, while lateral defects reflect localized disturbances in morphogenetic movements. The cardiogenic field, derived from the splanchnic mesoderm, undergoes a complex migration and folding process to form the primitive heart tube [116]. Disruptions to this process, especially when accompanied by incomplete ventral mesodermal fusion, can result in EC and other cardiac malformations [117]. Similarly, the diaphragm originates from several embryonic sources, including the septum transversum and the pleuroperitoneal folds. These structures depend on the coordinated development of the mesoderm. Disruption of these pathways provides a mechanistic explanation for the range of anomalies observed in CS [106]. Comparative embryology offers valuable insights into these conditions. In veterinary medicine, especially in canine models, EC has been systematically classified into different types [104], offering a structured framework that can be applied to human cases. This approach clarifies diagnostic differences between Cantrell’s syndrome and related conditions, such as body stalk anomaly (BSA), and highlights conserved embryological mechanisms across species. Within a One Health framework, cross-species analyses emphasize the interconnectedness of human and animal developmental biology. These analyses highlight the potential of comparative approaches to advance our understanding of congenital anomalies.

Thus, this review reexamines the existing literature on Cantrell’s syndrome through the lens of comparative development. Integrating veterinary classifications into the analysis of human cases aims to improve diagnostic accuracy, shed light on shared mesodermal pathways, and propose a unified framework for understanding the embryogenesis of thoracic and abdominal malformations.

## 2. CS Classification

The syndrome’s defining feature, EC, has been inconsistently classified, which limits the ability to compare cases and understand their embryological origins [104]. To address this issue, a structured classification system for midline and lateral defects has been proposed (Table 1). Midline defects include: umbilical thoracoabdominoschisis (UThAb) with an abnormal umbilical cord and omphalocele, supraumbilical thoracoabdominoschisis (SUThAb) with a normal umbilical cord, umbilical abdominoschisis with a diaphragmatic defect (UAb + DD) and an abnormal umbilical cord and omphalocele, supraumbilical abdominoschisis with a diaphragmatic hernia (SUAb + DD), supraumbilical incomplete central defect (SUICD), and umbilical hernia with a diaphragmatic defect (UH + DD). Lateral defects (LAb and gastroschisis) are further subdivided into thoraco-lateral abdominoschisis (ThLAb) and sternal lateral abdominoschisis (StLAb). Table 1 summarizes the distribution of body wall defects reported in human cases of Cantrell syndrome, as determined by a comprehensive literature review. The defects are grouped into midline and lateral categories, with midline anomalies representing most documented cases (153 out of 165 cases, 92.7%).

When these diverse anomalies are diagnosed as CS, the distinction between midline and lateral defects becomes blurred, and the syndrome is defined too broadly. This conflation can obscure the underlying mechanisms, as midline defects represent systemic failures of embryonic fusion, while lateral defects reflect localized folding errors [106]. It is crucial to recognize this difference. If all such situations are labeled as CS, the clinical and embryological specificity of the diagnosis is lost. This complicates comparisons across cases and hinders progress in understanding the syndrome’s true origins. Therefore, it is essential to carefully distinguish between CS and related anomalies to avoid diagnostic dilution and preserve the integrity of clinical and embryological analyses.

Midline anomalies represent most documented cases. Midline defects include umbilical cord–related and non–umbilical cord defects. These range from thoracic, abdominal and abdominal/omphalocele presentations to various supraumbilical abnormalities, such as supraumbilical thoracic and abdominal defects, isolated cord defects, and umbilical hernias. The most frequently described subtype is supraumbilical abdominal defects. In contrast, lateral defects are far less common and consist exclusively of non-umbilical cord anomalies, including thoracic defects, lateral thoracic and abdominal defects, and lateral abdominal defects. Overall, the table reflects the predominance of midline structural abnormalities in published human cases and highlights the anatomical variability documented in the literature. In veterinary medicine, particularly in dogs, EC has been documented and systematically categorized into distinct types [104]. This classification system can be applied to human cases, offering greater clarity in distinguishing CS from related anomalies, such as BSA. This framework integrates human and veterinary data from a One Health perspective.

## 3. CS in Human Medicine

CS is characterized by a combination of five midline defects that affect the abdominal wall, sternum, diaphragm, pericardium, and heart [1,105,108]. The clinical presentation can vary greatly, ranging from complete to partial or atypical forms, which complicates diagnosis. Although 165 cases have been documented, the heterogeneity of diagnostic criteria has limited the ability to establish standardized prognostic frameworks. Mortality remains high, especially in cases involving severe cardiac malformations, underscoring the necessity of more precise classification systems. Current approaches often group diverse anomalies under a single label, obscuring pathogenetic distinctions and complicating clinical management and developmental interpretation.

To clarify and standardize the analysis of CS, the 165 documented cases were reorganized into nine tables based on the morphology and topography of body wall defects, umbilical cord status, and associated anomalies. The cases in all tables are numbered chronologically according to their order of publication. When a publication reported more than one case, each case was assigned to and retained under the original author’s reference number. This ensured accurate case tracking and maintained precision when individual cases were cited or discussed later. In all the tables, symbols and terms in parentheses indicate the authors’ diagnostic interpretation based on the descriptions and photographs provided in the original publications. These annotations are used when the information available allows us to infer additional features, clarify the type of body wall defect, identify umbilical cord anomalies, or propose a diagnosis different from that stated by the reporting authors. Midline defects, representing the majority of cases, were subdivided into seven tables to capture the spectrum of supraumbilical, thoracic, abdominal, and umbilical ring presentations (Table 2, Table 3, Table 4, Table 5, Table 6, Table 7 and Table 8). Lateral defects, though rare, were grouped into a separate table to emphasize their distinctive asymmetric characteristics (Table 9). A ninth table was reserved for special cases that did not conform to traditional definitions, including those without body wall involvement or mixed supraumbilical–thoracic anomalies (Table 10). Separating the cases into these nine tables allows for a systematic comparison across subtypes. It also ensures transparent documentation of the original designations of the authors and our reclassifications. This approach underscores the heterogeneity of CS while maintaining a unified framework for interpretation. Table 2, Table 3, Table 4, Table 5, Table 6, Table 7 and Table 8 present the subtypes of midline defects, along with case details including gender, associated anomalies, and the authors’ and proposed diagnoses. Table 2 provides a summary of the details concerning thoracoabdominoschisis in cases where umbilical cord defects are present.

Table 2 presents seven cases of thoracoabdominoschisis (ThAb) associated with UCD. All cases exhibit full-thickness disruption of the midline extending from the thorax into the abdomen with exposure of cardiac structures and abdominal viscera. The presence of a UCD, such as a short cord, cysts, abnormal coiling, or a single umbilical artery, was confirmed through direct image review. These cases consistently demonstrate high rates of EC and complex intracardiac anomalies, which reinforces the severe phenotype associated with ThAb. Each entry includes the original author designation, our post hoc reclassification, umbilical cord status, type of cardiac exposure, and overlay tags for PC class and BSA type, when applicable. This subgroup represents the most extensive form of midline defect in the Cantrell spectrum, highlighting the diagnostic importance of cord morphology and thoracic and abdominal continuity.

The term BSA was originally used to describe human congenital malformations [118,119,120,121]. However, the formal BSA classification was first developed in pigs as a comparative model [122]. This framework was then applied to human cases [123] and later extended to other species, such as dogs and cats, in which similar patterns of ventral closing defects were observed [124,125]. This system recognizes eight major BSA types, and several additional complexes, such as the sternal body wall complex (STBWC), spinal body wall complex (SPBWC), spinal limb body wall complex (SPLBWC), and sternal spinal body wall complex (SSBWC) [126]. These complexes have already been applied to pigs, cats, and dogs to capture mixed constellations of sternal, spinal, and limb involvement [122,124,125]. In this review, we apply these complexes to human cases for the first time, providing a unified, comparative framework that aligns human and veterinary classifications. This approach reinforces the one health perspective by demonstrating that CS and related body stalk anomalies share conserved developmental pathways across species [104]. Table 2 shows that six of the seven cases of ThAb were diagnosed within the BSA framework. This finding highlights the significant overlap between CS and BSA phenotypes. A common feature across these cases was the presence of UCD, which appears to be a defining characteristic of this subgroup. The distribution included BSA Type VI with STBWC III, BSA Type V with SSBWC III, BSA Type V with SPLBWC III, and BSA Type II with STBWC I, and SPBWC III and ABS classifications. Most cases were assigned to PC Class 2, reflecting probable but incomplete pentalogy. Several cases showed EC or associated anomalies. This clustering indicates that ThAb with cord pathology tends to align with higher-order BSA types, in which ventral defects are accompanied by sternal, spinal, or limb involvement. These findings reinforce the diagnostic value of cord morphology in distinguishing severe BSA-related complexes. They also suggest that umbilical cord anomalies may be a unifying feature linking CS to the broader spectrum of BSA across species. Table 3 presents the characteristics of thoracoschisis with a normal umbilical cord.

Table 3 summarizes three cases of thoracoschisis (Th) in which the umbilical cord appeared normal with no evidence of structural anomalies, such as a single umbilical artery, cysts, abnormal coiling, or velamentous insertion. In this subgroup, Th is characterized by a full-thickness defect of the thoracic wall that is typically lateral or paramedian with variable degrees of cardiac exposure. Unlike UCD-positive Th, these cases demonstrate that severe thoracic wall disruption can occur independently of cord pathology. Each entry documents the author’s original designation, our post hoc reclassification, cord status, type of EC, and overlay tags for PC class and BSA type, when applicable. This small but distinct subgroup underscores the heterogeneity of CS, demonstrating that thoracic wall defects can present with normal cord morphology yet still involve significant cardiac pathology. Table 4 presents a summary of findings related to abdominoschisis with an umbilical cord defect.

Table 4 summarizes 19 cases of Ab in which umbilical cord anomalies were documented. Ab in this subgroup is characterized by a full-thickness midline abdominal wall defect and is frequently associated with abnormal cord morphology, such as a single umbilical artery, cysts, a short cord, or atypical coiling. These cord anomalies were confirmed through direct image review and serve as consistent markers of this phenotype. The table includes the original designations of the authors and our post hoc reclassification alongside details of cord status, type of EC (ExEC), and overlay tags for PC class and BSA type. The predominance of PC Class 2 assignments indicates partial or probable pentalogy, and several cases align with higher-order BSA complexes. Together, these cases highlight the strong link between Ab and umbilical cord pathology. This emphasizes the importance of cord anomalies in diagnosing severe forms of CS within the broader spectrum of BSA. In Table 4, which compiles 19 cases of Ab with umbilical cord anomalies, five were further classified within the BSA framework. These included BSA Type VIII with STBWC IV, BSA Type VII with SSBWC IV, and one case of BSA Type II with STBWC I. Most of these BSA-associated cases were assigned to PC Class 2 or 3, reflecting partial or incomplete pentalogy, while a smaller proportion fell into PC Class 1. Notably, EC was documented in several of these cases, reinforcing the severity of the phenotype. The clustering of Ab with cord pathology into higher-order BSA types highlights the strong developmental link between ventral wall disruption and umbilical cord anomalies. This subgroup demonstrates that cord anomalies are not incidental but rather integral markers of complex body stalk involvement, bridging CS with the broader comparative classification of ventral wall defects across species. Cases of supraumbilical thoracoabdominoschisis with a normal umbilical cord are detailed in Table 5.

Table 5 summarizes the 23 cases classified as SUThAb in which the umbilical cord was reported as normal. These cases serve as an essential comparison group for evaluating the role of cord anomalies in the pathogenesis and phenotypic variability of Ab. By isolating cases without cord pathology, the table provides a clearer assessment of the abdominal wall defect itself and helps distinguish primary SUThAb features from secondary changes associated with cord abnormalities. Individual case references are retained to ensure traceability and accuracy in subsequent discussion. Table 6 provides data on supraumbilical abdominoschisis with a normal umbilical cord.

Table 6 compiles 63 reported cases of SUAb in which the umbilical cord was described as normal. By excluding cases with associated cord anomalies, this dataset provides a clearer assessment of the intrinsic characteristics of the SUAb defect and allows for comparison with cases presenting umbilical cord pathology. The characteristics of supraumbilical incomplete central defects are summarized in Table 7.

Table 7 summarizes the 28 reported cases classified as a supraumbilical incomplete central defect (SUICD). These cases represent a distinct subgroup of supraumbilical abdominal wall defects, characterized by partial or incomplete disruption of the central supraumbilical region. Presenting these cases separately allows for a clearer delineation of their anatomical features and facilitates comparison with complete supraumbilical abdominoschisis (SUAb) and other related phenotypes. Table 8 summarizes cases of umbilical hernia.

Table 8 summarizes the ten reported cases diagnosed as umbilical hernias. The cases are presented separately to distinguish the true herniation of abdominal contents through the umbilical ring from the other congenital abdominal wall defects included in the review. Detailing this subset allows for a clearer comparison of anatomical features, associated findings, and clinical outcomes across the broader spectrum of umbilical and supraumbilical anomalies. Table 9 summarizes lateral abdominal wall defects.

Table 9 summarizes nine reported cases of lateral abdominal wall defects. These defects are characterized by an opening located lateral to the midline. This distinguishes them anatomically and developmentally from supraumbilical and central defects. Presenting these cases as a separate subgroup enables clearer comparisons of their morphological features, associated anomalies, and proposed pathogenetic mechanisms within the broader spectrum of abdominal wall defects. The special cases included in the review are presented in Table 10.

Table 10 includes three cases that were classified as “special cases” due to features that do not fit neatly into the main categories of abdominal wall defects analyzed in this review. The case reported by Angoulvant et al. [51] exhibits a diaphragmatic defect, a pericardial defect, and cardiac defects, such as an atrial septal defect and anomalous pulmonary venous return, but shows no body wall defect or umbilical cord defect. However, the absence of ventral body wall involvement suggests a more appropriate diagnosis of congenital heart disease with associated midline structural defects rather than incomplete PC. Similarly, the case described by Hubbard et al. [85] lacks a body wall defect and UCD but presents with a sternal defect; multiple cardiac defects, including a ventricular septal defect, single coronary artery, and atrial septal defect; and an external EC and additional anomalies, such as an encephalocele, craniofacial dysmorphism, and a cleft palate. Although the authors labeled it as PC, the constellation of findings aligns more closely with EC, accompanied by broader craniofacial and thoracic abnormalities. The third case, from Martadiansyah et al. [103], includes an umbilical incomplete central defect, a sternal defect, diaphragmatic defect, and patent ductus arteriosus, and significant cardiac defects. Although it is described as EC complicated by PC, the pattern of anomalies is more consistent with PC, specifically Class 1 in association with a body stalk anomaly (BSA) Type VIII, which corresponds to STBWC IV. Together, these cases demonstrate how overlapping phenotypes, particularly when UCDs, craniofacial anomalies, or lateralized defects are present, blur the distinction between PC and other embryologically distinct processes, highlighting the need for clearer differentiation.

## 4. Veterinary Perspective: Ectopia Cordis and Cantrell’s Syndrome

In contrast, veterinary medicine has advanced a systematic classification of BSA in pigs, dogs and cats [122,124,125,126], and EC in dogs, distinguishing cases by anatomical location and associated thoracic and abdominal defects for EC and skeletal structural defects for BSA. This structured approach provides clarity in differentiating between variations in presentation and embryological origin. Importantly, canine cases represent naturally occurring models of rare congenital anomalies, offering insights into mesodermal development and ventral body wall formation. These observations highlight the role of comparative embryology, as dogs provide a biologically relevant framework for understanding anomalies that mirror human conditions. In all the tables, symbols and terms in parentheses indicate the authors’ diagnostic interpretation based on the descriptions and photographs provided in the original publications. These annotations are used when the information available allows us to infer additional features, clarify the type of body wall defect, identify umbilical cord anomalies, or propose a diagnosis different from that stated by the reporting authors.

A retrospective descriptive analysis was performed on 19 published cases of congenital thoracic, abdominal and cardiac anomalies in dogs and cats historically associated with Pentalogy of Cantrell (PC) or related midline developmental defects. The presence or absence of the five classic PC components (abdominal wall defect, sternal defect, diaphragmatic defect, pericardial defect, and intracardiac anomalies) was extracted for each case, along with additional malformations, such as ectopia cordis, limb defects, craniofacial anomalies, and body stalk abnormalities. The reported diagnoses from the original authors were then compared to a standardized reclassification using contemporary PC criteria (classes 1–3) and complementary systems, including BSA types and STBWC/SSBWC categories. We recorded species, sex, and defect combinations to identify patterns, misclassifications, and phenotypic clusters. A summary of carnivore cases, their classification, and the proposed diagnoses is presented in Table 11.

Table 11 summarizes nineteen reported cases of congenital thoracic, abdominal and cardiac malformations in dogs and cats that fall within the spectrum of Pentalogy of Cantrell (PC) and related midline defects. For each case, the table lists the presence or absence of the five classic PC components: abdominal wall, sternal, diaphragmatic, pericardial, and cardiac defects. It also lists additional anomalies, such as ectopia cordis, limb defects, craniofacial defects, and body stalk abnormalities. The table also compares the original diagnosis given by each author with a standardized reclassification using current PC criteria. Overall, the table shows that most animals have multiple midline defects. Incomplete PC is the most common form, while the most severe cases—often those with ThAb—meet the criteria for complete PC. The table highlights the wide phenotypic variability of these conditions and illustrates how modern classification systems can more accurately reinterpret earlier case reports. Table 12 provides a summary of porcine cases, their classification, and the proposed diagnoses.

Table 12 summarizes six cases of porcine congenital malformations consistent with Pentalogy of Cantrell (PC) reported by Martín-Alguacil and Avedillo [105]. Each piglet exhibited a remarkably uniform pattern of defects beginning with ThAb as the primary body wall abnormality. This severe midline disruption is accompanied by consistent umbilical cord abnormalities, including short cords, abnormal coiling patterns (ACP), dispersed umbilical vessels (DUV), and, in some cases, single or hypoplastic umbilical arteries (SUA or HUA). All cases exhibit the five classical components of PC: body wall defect, sternal defect, diaphragmatic defect, pericardial defect, and intracardiac anomalies. These cases fulfill the criteria for PC Class 1 (complete PC). Cardiac defects vary among individuals and include atrial septal defects (ASD), ventricular septal defects (VSD), globular heart morphology (GHM), hypoplastic auricles, a single coronary artery, and severe anomalies, such as transposition of the great arteries (TGA) and mitral valve atresia (MAV). All piglets also present with ectopia cordis, which is an external manifestation of the most severe PC phenotypes. Additional visceral anomalies, such as ectopic caecum (EcC), ectopic liver (EcL), and amorphous liver masses (LAM), reinforce the profound disruption of ventral midline development. The proposed diagnosis consistently reclassifies all six cases as PC Class 1, accompanied by BSA Type VI and STBWC Type III, reflecting extensive involvement of the thoracic, abdominal, and umbilical structures. These uniform classifications indicate that these piglets exhibit a consistent and severe expression of the Cantrell spectrum with overlapping BSA features. Table 13 presents a summary of ruminant cases, their classification, and the proposed diagnoses.

Table 13 summarizes 16 cases of ruminants—mostly calves and two lambs—with congenital midline defects involving the thoracic region. There is a strong predominance of ectopia cordis (EC). Unlike pigs and carnivores, in which pentalogy of Cantrell (PC) is common, the ruminants in this dataset exhibit a distinct pattern dominated by cervical or cervico-pectoral EC, with minimal or absent involvement of the abdominal wall. Nearly all cases exhibit an absent body wall defect, and the umbilical cord is either normal or not reported. This indicates that these anomalies primarily affect the upper thoracic and cervical midline rather than the abdominal region. Every case in the table exhibits sternal defects and external ectopia cordis, confirming a consistent failure of thoracic midline closure. Many animals also exhibit pericardial defects and complex cardiac malformations, such as double apex, duplicated cranial vena cava, ventricular septal defects, anomalous pulmonary venous return, a single coronary artery, and a double-outlet right ventricle. These cardiac anomalies are often accompanied by nonstructural spinal defects, cleft palate, colonic stenosis, and visceral abnormalities, such as hepatic fibrosis or amorphous liver masses. These abnormalities reflect broader disruptions of embryonic midline development. Most cases were originally diagnosed as cervical, cervico-pectoral, or thoracic ectopia cordis, and the proposed diagnosis confirms this interpretation. Only one case (Case 38) meets the criteria for PC Class 2 due to the presence of an umbilical hernia, a diaphragmatic defect, a pericardial defect, and multiple intracardiac anomalies. All other cases lack the abdominal wall component required for PC and are classified as ectopia cordis (EC). Overall, the table shows that ruminants have a typical EC-dominant phenotype with sternal defects and severe cardiac malformations, but not the abdominal wall defects that are common in PC. These characteristics distinguish ruminant presentations from those of pigs and carnivores, suggesting species-specific patterns in ventral midline developmental failure.

## 5. Comparative Analysis: Applying Veterinary Classification to Human Cases

Applying the canine and pig classification system to human cases reveals that several anomalies historically labeled as CS align more closely with BSA. This reclassification suggests that CS and BSA may represent points along a continuum of malformative processes rather than discrete syndromes. Recognizing this continuum is critical for refining diagnostic accuracy and avoiding conflation of distinct pathogenetic mechanisms. Comparative analysis thus underscores the value of veterinary models in sharpening human diagnostic frameworks and clarifying the developmental variability observed across cases.

A combined analysis of 165 human cases and veterinary data from carnivores, pigs, and ruminants shows that CS and other ventral midline defects form a continuous spectrum of developmental disruption across species, though there are clear species-specific patterns. In humans, stratifying cases across Table 2, Table 3, Table 4, Table 5, Table 6, Table 7, Table 8, Table 9 and Table 10 reveals that the severity and anatomical extent of the defect correlate strongly with umbilical cord morphology. ThAb with cord anomalies (Table 2) is the most severe condition on this spectrum. It is characterized by full-thickness thoracic and abdominal disruption, external ectopia cordis, and complex intracardiac defects. These cases frequently correspond to higher-order BSA types and sternal-spinal-limb complexes, similar to the porcine model in which all reported piglets exhibit ThAb, abnormal umbilical cords, and complete PC. Abdominoschisis with cord anomalies (Table 4) follows a similar pattern at the abdominal level. There is strong clustering into BSA types VII–VIII and II, and predominant assignment to PC class 2 or 3. Conversely, thoracoschisis with normal umbilical cords (Table 3), SUThAb and SUAb with normal umbilical cords (Table 5 and Table 6), and SUICD (Table 7) demonstrate that significant thoracic or abdominal wall defects can occur independently of cord pathology and typically manifest as milder or more localized expressions of the Cantrell/BSA field.

The carnivore dataset closely parallels human distribution. Like humans, dogs and cats exhibit the full range of phenotypes, from complete PC with ThAb and cord anomalies (analogous to human Table 2) to incomplete PC and SUICD-like presentations (resembling human cases in Table 5, Table 6, Table 7 and Table 8). As in humans, ThAb in carnivores is strongly associated with severe cardiac defects, sternal agenesis, and high-order BSA classifications. Cases with normal umbilical cords, on the other hand, tend to fall into PC Class 3 or remain outside the PC spectrum. This alignment reinforces developmental continuity between human and carnivore presentations, supporting the use of BSA and STBWC/SSBWC complexes as comparative tools across species.

Pigs occupy a unique position within this comparative framework. All six porcine cases exhibit a highly uniform and extreme phenotype consisting of ThAb, severe umbilical cord anomalies, external ectopia cordis, and complex intracardiac malformations. These cases are consistently classified as PC Class 1 with BSA Type VI and STBWC III. This homogeneity contrasts with the broader phenotypic variability seen in humans and carnivores, suggesting that pigs express a particularly severe and stable form of ventral midline defects. Notably, the BSA classification was initially developed in pigs, subsequently applied to humans, and then to carnivores [122,123,124,125,126]. The porcine data in this review reaffirm the value of this system for capturing high-order, multisystem involvement.

By contrast, ruminants display a distinct, largely non-abdominal phenotype. Calf and lamb cases are characterized by cervical, cervicothoracic, or thoracic ectopia cordis, along with sternal defects and complex cardiac anomalies, though there is no abdominal wall disruption or umbilical cord pathology. These cases do not align well with the human ThAb, Ab, SUAb, or SUICD groups. Instead, they resemble a small subset of human thoracoschisis cases with normal umbilical cords (Table 3). The consistent cranial displacement of the defect in these cases suggests a species-specific vulnerability of the upper thoracic and cervical midline. This distinguishes ruminants from the thoracic, abdominal and umbilical patterns seen in humans, pigs, and carnivores.

Taken together, these findings highlight two major axes that define the comparative expression of Cantrell-related defects across species: the craniocaudal level of the ventral defect and the presence or absence of umbilical cord anomalies. Humans, pigs, and carnivores share a common pattern: ThAb or Ab combined with cord pathology marks the most severe BSA-associated phenotypes, while defects with normal umbilical cords tend to be milder or anatomically restricted. Ruminants, however, cluster into a separate ectopia cordis phenotype that is focused cranially and minimally involves the umbilical region. This comparative perspective reinforces the one health concept by demonstrating that CS, BSA, and related ventral defects arise from conserved developmental pathways, yet manifest differently depending on species-specific embryologic constraints. It also underscores the diagnostic value of umbilical cord morphology as a cross-species marker of high-order body stalk involvement and provides a unified framework for interpreting human and veterinary cases within a shared developmental continuum.

## 6. Embryological Insights and Pathogenetic Mechanisms

CS is characterized by a range of midline defects affecting the thoracic and abdominal walls, sternum, diaphragm, pericardium and heart [1,104,105,108]. Understanding its embryological origins is crucial for grasping why midline defects, and less commonly lateral defects, can occur. From an embryological perspective, ventral body wall anomalies represent a spectrum of developmental failures that occur at different stages and through distinct mechanisms. Omphalocele arises when the physiological herniation of the midgut, which usually occurs during weeks 6–10 of human embryogenesis, does not resolve properly, resulting in abdominal contents herniating into the umbilical cord within a membranous sac. In contrast, supraumbilical midline defects, as seen in Cantrell’s syndrome, originate much earlier—between human days 14–18 and canine days 14–35—when the lateral plate mesoderm fails to fuse at the ventral midline [104,106]. This produces systemic anomalies involving the sternum, diaphragm, pericardium and abdominal wall. Within this same developmental window, sternal defects result from the incomplete fusion of paired sternal bars derived from the somatic mesoderm [143]. This leads to clefts or agenesis of the sternum. Diaphragmatic defects, meanwhile, reflect the abnormal migration and incorporation of the septum transversum and pleuroperitoneal membranes [144,145]. This produces anterior diaphragmatic gaps that often accompany the Cantrell spectrum. Gastroschisis is characterized by a localized disruption to the folding of the lateral body wall around weeks 4–6 in humans, typically just to the right of the umbilicus [114]. This results in a paraumbilical opening without a covering sac and is usually not associated with cardiac or diaphragmatic anomalies. Finally, rectus diastasis is a milder defect of ventral body wall development caused by incomplete fusion of the linea alba, which is derived from the lateral plate mesoderm [146,147]. Unlike the other anomalies, it does not involve a true wall defect or herniation but rather manifests as a separation of the rectus muscles along the midline. Taken together, these conditions demonstrate how disturbances in mesodermal fusion, folding and midgut migration can generate a range of thoracic and abdominal malformations, from severe open defects to subtle connective tissue abnormalities, in both human and canine embryogenesis.

In vertebrates, the body wall comprises the skin, muscles, and supportive connective tissues. Its formation depends on a series of tightly regulated, sequential events during embryonic development [106]. The formation of the two body cavities and the sealing of the body wall depend on the coordinated interaction of numerous developmental processes. Disruption to these processes during embryogenesis can result in serious structural anomalies in newborns, including congenital diaphragmatic hernia and ventral body wall defects such as gastroschisis and omphalocele [106,114]. To understand this process, we present a detailed overview of the essential mechanisms for the correct development of the abdominal and thoracic walls. This analysis offers valuable insights into body wall formation and, importantly, clarifies the embryological differences between lateral and midline defects. Following fertilization, the zygote undergoes cleavage and compaction to form the blastocyst. After compaction, the morula develops into a blastocyst, losing its totipotent capacity in the process [106]. The inner cell mass gives rise to the embryoblast, while the outer layer differentiates into the trophoblast. The trophoblast supports implantation into the endometrium and provides nutrition. Within the embryoblast, two distinct cell populations emerge: the epiblast, which is positioned next to the amniotic cavity, and the hypoblast, which is oriented towards the blastocyst cavity. Amnioblasts lie adjacent to the trophoblast and remain continuous with the epiblast. The epiblast cells are arranged radially and become enclosed by the amniotic cavity. Meanwhile, the hypoblast (visceral endoderm) cells delaminate from the epiblast and are separated by a basal lamina. They subsequently line the secondary yolk sac. The establishment of these two layers—the epiblast and the hypoblast—defines the embryo’s dorsoventral axis. During gastrulation, the initially two-dimensional structure remodels into a three-dimensional trilaminar disk, ultimately forming the three germ layers. By the end of the second week, the primitive streak appears, marking the beginning of further morphogenetic events, as it does in dogs at a comparable stage [148,149,150]. This marks the start of gastrulation, which results in the formation of a trilaminar embryo. The notochord then directs neurulation and somite differentiation. The rapid expansion of the somites and the lateral plate mesoderm initiates the folding process, incorporating the yolk sac into the embryonic body and establishing the common body cavity [146]. By around week 3 in humans and day 20 in dogs, the umbilical cord and connecting stalk begin to develop [150]. By week 7 in humans and around day 30 in dogs, the cord is fully formed and takes on metabolic functions. The pleuroperitoneal folds then begin to fuse between weeks 4 and 6, with complete closure of the pleuroperitoneal canals occurring by the end of week 7. In dogs, the equivalent process occurs between days 20 and 35 of embryogenesis, with fusion of the pleuroperitoneal folds and closure of the canals completed by approximately day 35 [124]. In humans, the transverse septum emerges around day 22 of embryogenesis. Physiological herniation of the intestine normally occurs by week 6 in humans and day 30 in dogs, resolving by week 10 in humans and day 35 in dogs [151,152]. If this retraction fails, an omphalocele develops. Conversely, rupture of the amnion between weeks 8–10 in humans or days 30–35 in dogs leads to gastroschisis [115]. During the early fusion window (days 14–18 in humans and days 14–35 in dogs), disruption to the fusion of the mesoderm can result in supraumbilical midline defects, sternal defects, diaphragmatic defects, pericardial defects and rectus diastasis. These anomalies collectively define the spectrum of CS, representing failures of early ventral body wall formation. In contrast, omphalocele and gastroschisis arise later, during the stages of intestinal herniation and body wall closure.

**Figure 1:**
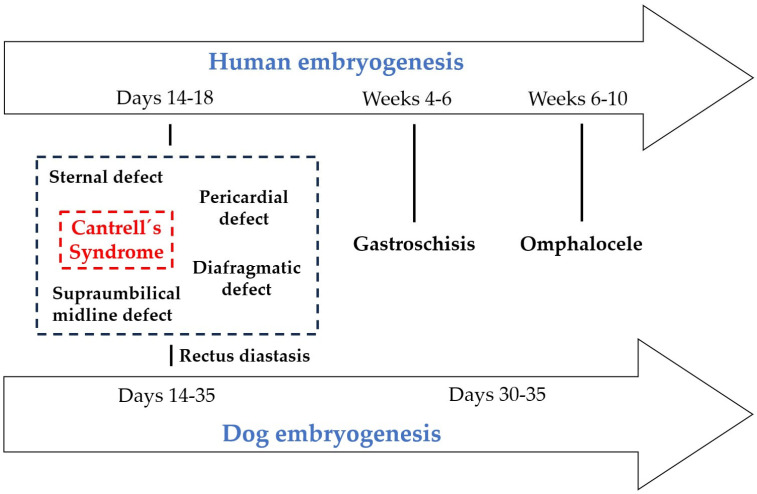
Critical stages of ventral body wall development, showing the timing of these stages in embryos and the corresponding anomalies observed in humans and dogs.

The midline and lateral body wall defects arise from disruptions in the complex morphogenetic processes that shape the ventral body wall during early embryogenesis. They represent a spectrum of anomalies—including omphalocele, gastroschisis, ectopia cordis, and bladder exstrophy—that reflect failures in midline fusion or lateral folding of the embryonic body wall [106]. As shown in Figure 1, the critical windows of ventral body wall development define the embryonic stages at which defects such as Cantrell’s spectrum, gastroschisis and omphalocele may occur.

Evidence from different species suggests that disruptions in the development of the lateral plate mesoderm represent the main mechanism underlying Cantrell’s syndrome and related thoracic and abdominal anomalies [104,105,108,153]. During gastrulation, mesodermal cells migrate and differentiate into the lateral plate mesoderm, contributing to the ventral body wall, diaphragm, pericardium and cardiogenic field. The prevailing theory places this critical period between days 14–18 of human embryogenesis [154,155], which corresponds to approximately days 14–35 in canine development. During this time, mesodermal folds must migrate and fuse towards the ventral midline [78]. Failures in this fusion process result in systemic midline defects affecting the sternum, diaphragm, pericardium and abdominal wall [104,105,108,153,154,155]. In contrast, localized disruptions to body wall folding generate lateral anomalies such as gastroschisis, which typically do not involve the heart or diaphragm. Further explanations for the occurrence of ectopia cordis and associated cardiac malformations lie in perturbations in cardiogenic field migration and folding [156,157]. Additionally, defective development of the septum transversum, which normally contributes to the formation of the diaphragm and the pericardium, exacerbates these anomalies. Taken together, human and animal embryological evidence highlights how a narrow developmental window of lateral plate mesodermal activity governs the range of thoracic and abdominal malformations observed in different species. Table 14 presents a comparative embryological overview of ventral body wall defects in humans and dogs.

Table 14 offers a side-by-side comparison of the embryological pathways that lead to ventral body wall defects in humans and dogs, highlighting their similarities and differences. It summarizes key developmental processes, including midline folding, sternal and diaphragmatic formation, cardiac descent, and umbilical ring closure, and maps them onto the specific defects observed in each species. By comparing the timing of embryonic disruption, the anatomical structures affected, and the resulting characteristic phenotypes, the table highlights conserved mechanisms underlying Cantrell-related anomalies and illustrates species-specific differences in expression. This summary helps readers understand how similar developmental failures can produce parallel patterns of thoracic and abdominal defects in humans and dogs. It also reinforces the value of comparative embryology in interpreting complex ventral wall defects.

Omphaloceles result from continued physiological midgut herniation. The displaced intestine fails to return to the abdominal cavity, ultimately causing intestinal malrotation and abnormal positioning [106,114]. Gastroschisis is a congenital structural abnormality of the abdominal wall, characterized by the extrusion of visceral organs through a paraumbilical defect [106,114,115]. Unlike omphalocele, the herniated intestine lacks an amniotic covering and is therefore directly immersed in amniotic fluid [110]. Several pathogenetic mechanisms have been proposed to explain its origin over the past decades: impaired mesodermal development [157,158]; rupture of the amnion adjacent to the umbilical ring [110]; estrogen-induced thrombosis of the umbilical vein [111]; malformation of the right vitelline artery [112]; and defective invagination of the secondary yolk sac and omphalomesenteric duct, despite normal abdominal wall formation otherwise [113].

The convergence of human and canine data highlights conserved developmental pathways and emphasizes the importance of comparative embryology in congenital anomaly research. By integrating veterinary and human findings, a unified framework emerges that links mesodermal morphogenetic failures to the spectrum of thoracic and abdominal malformations. This perspective advances both clinical and developmental biology by situating Cantrell’s Syndrome within broader embryological processes rather than treating it as an isolated clinical entity.

## 7. Discussion

CS remains a rare and complex anomaly with significant heterogeneity in clinical presentation and embryological interpretation [99,102]. The comparative approach adopted here, which involves applying veterinary classifications of ectopia cordis and body wall defects to human cases, provides new insights into diagnosing and categorizing this syndrome. In both humans and animals, CS is fundamentally linked to the complex process of body cavity closure [105,106]. The variety of ways in which CS presents clinically reflects the points at which these developmental events can be disrupted. Failures in mesodermal fusion, ventral folding, or incorporation of the septum transversum result in a range of anomalies that define Cantrell’s pentalogy [84,99,108]. Understanding these embryological foundations clarifies the variability of the syndrome and provides a framework for distinguishing it from related ventral body wall malformations. The literature fully supports the theory that CS results from a failure of the lateral plate mesoderm to migrate and fuse at the ventral midline during early embryogenesis [157,158,159]. This mechanism can explain why midline defects are so common, since the sternum, diaphragm, pericardium, abdominal wall, and heart all originate from the ventral mesodermal field. The rare occurrence of lateral defects suggests that the embryological insult may sometimes be more extensive or involve adjacent developmental fields [158]. The comparative analysis of human and veterinary cases presented in this review sustains this idea, showing that across species, the severity and anatomical distribution of ventral body wall defects consistently reflect the timing, location, and extent of mesodermal disruption. The animal data fully endorse this embryological model. In carnivores, for instance, ThAb, accompanied by UCD, closely resembles the most severe human cases. Dogs and cats exhibit the full range of phenotypes, from complete PC with ThAb and sternal agenesis to complex cardiac anomalies, to incomplete forms resembling human SUICD and supraumbilical defects. These parallels reinforce the idea that the same ventral mesodermal field is vulnerable across species and that the presence of umbilical cord anomalies reliably indicates high-order body stalk involvement. Porcine cases offer an especially striking point of comparison. All affected piglets display a highly uniform and severe phenotype characterized by ThAb, severe umbilical cord abnormalities, external ectopia cordis, and complex intracardiac malformations. These cases consistently fall within PC Class 1 and correspond to BSA Type VI [105]. This remarkable homogeneity suggests that pigs exhibit an especially severe and consistent form of ventral midline disruption, making them a powerful model for understanding the upper end of the Cantrell/BSA spectrum. In contrast, ruminants exhibit a distinct cranial phenotype, with cervical or cervicothoracic ectopia cordis accompanied by sternal and cardiac defects, but with minimal abdominal involvement and an absence of umbilical cord pathology [137,138,139,140,141,142]. This pattern resembles only a small subset of human thoracoschisis cases, highlighting species-specific differences in craniocaudal susceptibility of the ventral midline. One valuable contribution of the canine model is its classification of ectopia cordis types, enabling more precise differentiation of cases that would otherwise be broadly grouped under CS. A reevaluation of 165 human cases revealed that several were more accurately categorized as BSA, highlighting the need for a unified cross-species framework. The porcine BSA classification, initially developed in pigs and subsequently applied to humans, dogs, and cats, further reinforces this integrative approach [122,123,124,125,126]. Together, these comparative systems clarify the embryological mechanisms involved and emphasize mesodermal developmental defects as a shared pathogenetic pathway.

In recent years, reports of abnormalities in the formation of the abdominal cavity and wall have increased, yet the physiological and pathophysiological mechanisms underlying these malformations remain incompletely understood. Current evidence suggests that epigenetic influences play a significant role, while chromosomal abnormalities account for only a small percentage of cases [106]. Understanding the chronological, spatial, and morphogenetic progression of organogenesis is essential to appreciating how intrinsic and extrinsic disruption affect organ system differentiation [106]. Adopting a One Health perspective strengthens this analysis by framing congenital anomalies as a shared developmental vulnerability across species. Veterinary data, which are often underutilized in human medicine, provide valuable comparative models for rare syndromes. Canine ectopia cordis, for example, offers insights directly applicable to human cases, and the porcine BSA classification enhances diagnostic precision and deepens our understanding of embryological mechanisms. Together, these models bridge gaps in classification and diagnosis, showing how veterinary embryology can inform human clinical practice and vice versa.

In his original description, Cantrell emphasized a supraumbilical midline defect as a defining hallmark of the syndrome, reflecting a specific embryologic failure of the ventral body wall during early thoracic and abdominal development [1]. However, as more human cases were documented, clinicians and researchers recognized that the range of midline abnormalities was broader than initially proposed (Table 5, Table 6 and Table 7). Additional defects, some of which were umbilical or variably positioned along the midline, were gradually accepted as part of the syndrome’s phenotypic range (Table 4). Recently, some authors have included lateral body wall defects despite their distinct embryologic origins and later timing in embryonic development (Table 9). This raises questions about whether these anomalies arise from the same pathogenic mechanism. The inclusion of body stalk anomalies, particularly when the umbilical cord is malformed or absent, further complicates matters, as these defects stem from an even earlier and more global disruption of embryonic folding. Taken together, the expanding list of associated defects suggests that what has been grouped under “Cantrell syndrome” may actually represent multiple developmental processes with overlapping but not identical pathways, rather than a single, unified entity. Therefore, it may be time to reconsider the classification and distinguish these processes more clearly to improve diagnostic precision and better understand the underlying embryologic mechanisms. Nevertheless, limitations must be acknowledged. The number of documented veterinary cases is relatively small compared to human reports, and species-specific embryological differences may prevent direct extrapolation. The retrospective nature of case analysis introduces variability in diagnostic criteria and reporting standards. These challenges underscore the necessity of prospective, standardized studies in both veterinary and human medicine to validate the proposed comparative framework. Despite these limitations, using animal models to compare the classification of Cantrell’s syndrome represents a constructive step forward. It shows how veterinary findings can enrich human medicine, encourages adopting cross-species perspectives in congenital anomaly research, and paves the way for future interdisciplinary studies.

This review provides a unified, cross-species framework for understanding CS and related body wall defects. However, several limitations must be acknowledged. First, the available human cases vary in quality, terminology, and diagnostic detail, which may introduce classification bias despite careful reevaluation. Second, veterinary reports vary widely in completeness, particularly regarding umbilical cord morphology and intracardiac findings. This limits direct comparison across species. Third, the rarity of these anomalies means sample sizes, especially in non-human species, remain small, reducing the ability to draw firm epidemiological conclusions. Finally, embryological interpretations rely on published descriptions rather than standardized imaging or histopathology, constraining the precision of developmental inferences. These limitations underscore the necessity of more systematic, multidisciplinary documentation of ventral body wall defects in human and veterinary medicine.

Future research on congenital anomalies should prioritize developing a harmonized classification system for ectopia cordis and related ventral body wall defects that can be applied across species. This framework must integrate veterinary and medical perspectives to ensure consistent terminology and diagnostic criteria. To advance this goal, close collaboration is required among veterinarians, physicians, embryologists, and geneticists. This collaboration will foster truly comparative research that bridges species boundaries and deepens our understanding of shared developmental mechanisms. Systematically collecting prospective data using standardized diagnostic criteria across human and veterinary medicine will reduce variability and strengthen the reliability of case documentation. Concurrently, embryological research must expand to investigate mesodermal developmental defects as a shared pathogenetic pathway, utilizing animal models to supplement human studies. Integrating these efforts into the One Health framework emphasizes congenital anomalies as a shared challenge across species and ensures that rare syndromes benefit from cross-species insights. Ultimately, translating these comparative findings into clinical applications could lead to improved diagnostic protocols and earlier detection strategies in both human and veterinary medicine, and potentially preventive measures.

## 8. Conclusions

This review shows that CS and other ventral body wall defects are part of a single spectrum of midline developmental disorders caused by disruptions in the ventral mesoderm during early embryogenesis. By integrating 165 human cases with comparative data from dogs, cats, pigs, and ruminants, we demonstrate that the embryological mechanisms underlying these anomalies are conserved across species despite varying anatomical expression. The human dataset reveals clear stratification of phenotypes based on defect location and umbilical cord morphology. ThAb and abdominoschisis, accompanied by umbilical cord anomalies, represent the most severe body-stalk-associated forms. Carnivores closely mirror this distribution, while pigs consistently express an extreme, uniform phenotype that aligns with complete CS and high-order BSA types. In contrast, ruminants exhibit a distinct cranial pattern dominated by cervical and cervicothoracic ectopia cordis, which highlights species-specific differences in ventral midline vulnerability. Together, these findings underscore the importance of a comparative, cross-species approach to understanding the embryological origins and phenotypic variability of Cantrell-related anomalies. Veterinary models, particularly the canine ectopia cordis classification and the porcine BSA system, provide powerful tools for refining human diagnoses and clarifying the developmental pathways involved. This One Health approach emphasizes that congenital ventral body wall defects are not limited to human medicine, but rather reflect shared biological processes across mammals. Future progress will depend on standardized, prospective data collection and deeper interdisciplinary collaboration among clinicians, veterinarians, embryologists, and geneticists. These efforts will improve diagnostic accuracy, enable earlier detection, and ultimately enhance outcomes for individuals affected by these rare yet clinically significant malformations.

## Figures and Tables

**Table 1:** Classification of Body Wall Defects in Cantrell’s Syndrome.

Category	Type	Definition	Umbilical Cord	Associated Defects	Total Cases	Prevalence *n* = 165
Midline Defects	UThAb	Umbilical thoracoabdominoschisis	Abnormal (omphalocele)		7	4.24%
	UAb + DD	Umbilical abdominoschisis	Abnormal (omphalocele)	Diaphragmatic defect	19	11.51%
	SUThAb	Supraumbilical thoracoabdominoschisis	Normal		23	13.93%
	Th	Thoracoschisis	Normal	Sternal defect	3	1.81%
	SUAb + DD	Supraumbilical abdominoschisis	Normal	Diaphragmatic hernia	63	38.18%
	SUICD	Supraumbilical incomplete central defect	Normal	Sternal defect	28	16.97%
	UH + DD	Umbilical hernia	Normal	Diaphragmatic defect	10	6.06%
Lateral Defects	LTHAb	Lateral thoracoabdominoschisis	Normal		3	1.81%
	LTh	Lateral torachoschisis	Normal		2	1.21%
	Lab	Lateral abdominoschisis	Normal (gastroschisis)		4	2.42%

**Table 2:** Umbilical Thoracoabdominoschisis (UThAb) with Umbilical Cord Defects (*n* = 7).

References	Case/Gender	BWD	UCD	StD	DD	PD	CD	ExEC	OD	Author’s Diagnosis	Proposed Diagnosis
[2]	Case 1 ♀	ThAb	∅ (+)	∅	+	+	AAA	Type 1	Ee, AE, Cch, CA, St-SpD	Ee in Cantrell-Haller-Ravitsch Syndrome	PC Class 2 BSA Type VI SPBWC III
[6]	Case 17 ♂	ThAb	+ SUA	+	+	∅	PDA, MVA, ASD	Type 1	Ee, CL, CP, ABS	PC with Ee and ABS	PC Class 2 BSA Type VI STBWC III ABS
[64]	Case 113 ∅	O (ThAb)	∅ (+)	+	+	∅	VSD	Type 3	Ee, St-SpD	PC with Ee and SpDs	PC Class 2 BSA Type V SSBWC III
[68]	Case 119 ♀	(ThAb)	∅ (+)	+	∅	∅	∅	Type 2	HR, St-LD, St-SpD	PC associated with LD	BSA Type V SPLBWC III
[69]	Case 120 AG	SUAb (ThAb)	∅ (+)	+	+	∅	∅	Type 1	AA, St-GuD, NSt-LD	PC	BSA Type II STBWC I
[89]	Case 146 ♂	ThAb	+ SUA Cyst	∅	∅	∅	∅	+	CL, CP, St-LD	ThAbEC	EC
[97]	Case 158 C1, ♀	ThAb	+	+	+	∅	∅	Type 1	∅	PC	BSA Type VI STBWC III

∅, not reported; **AA**, anal atresia; **AAA**, aplasia of the aortic arch; **ABS**, amniotic band syndrome; **AE**, adrenal ectopia; **ASD**, atrial septal defect; **BSA**, body stalk anomaly; **BWD**, body wall defects; **CA**, cerebellar aplasia; **Cch**, cranioschisis; **CD**, cardiac defects; **CL**, cleft lip; **CP**, cleft palate; **DD**, diaphragmatic defect; **EC**, *Ectopia cordis*; **Ee**, exencephaly; **ExEC**, external *ectopia cordis*; **HR**, hypoplastic ribs; **LD**, limb defect; **MVA**, mitral valve agenesis; **NSt-LD**, non-structural limb defect; **O**, omphalocele; **OD**, other defects; **PC**, pentalogy of Cantrell; **PD**, pericardial defect; **PDA**, patent ductus arteriosus; **SPBWC**, spinal body wall complex; **SpDs**, spinal dysraphism; **SPLBWC**, spinal limb body wall complex; **SSBWC**, sternal spinal body wall complex; **StD**, sternal defect; **STBWC**, sternal body wall complex; **St-GuD**, genitourinary defects; **St-LD**, structural limb defect; **St-SpD**, structural spinal defect; **SUA**, single umbilical artery; **ThAb**, thoracoabdominoschisis; **ThAbEC**, toraco-abdominal *ectopia cordis*; **UCD**, umbilical cord defect; **VSD**, ventricular septal defect.

**Table 3:** Thoracoschisis (Th) with Normal Umbilical Cord (*n* = 3).

References	Case/Gender	BWD	UCD	StD	DD	PD	CD	ExEC	OD	Author’s Diagnosis	Proposed Diagnosis
[21]	Case 37 C4, ∅	Th	-	+	∅	+	∅	Type 3	∅	PC	PC Class 3
[80]	Case 137 ♀	Th	-	+	∅	+	∅	Type 3	∅	EC	EC
[90]	Case 154 ♀	Th	-	+	+		ASD, VSD	Type 3	BCL, CP, CrfD, ABS	PC	PC Class 2

**∅**, not reported; **ABS**, amniotic band syndrome; **ASD**, atrial septal defect; **BWD**, body wall defects; **BCL**, bilateral cleft lip; **CD**, cardiac defects; **CP**, cleft palate; **CrfD**, craniofacial dysmorphism; **DD**, diaphragmatic defect; **EC**, *Ectopia cordis*; ExEC, external ectopia cardiaca; **OD**, other defects; **PC**, Pentalogy of Cantrell; **PD**, pericardial defect; **StD**, sternal defect; **Th**, thoracoschisis; **UCD**, umbilical cord defect; **VSD**, ventricular septal defect.

**Table 4:** Umbilical Abdominoschisis (UAb) with Umbilical Cord Defect (*n* = 19).

References	Case/Gender	BWD	UCD	StD	DD	PD	CD	ExEC	OD	Author’s Diagnosis	Proposed Diagnosis
[3]	Case 3 S.D., ♂	O (Ab)	+	∅	+	+	TF	Type 1	∅	PC	PC Class 2
	Case 4 F.M., ♀	O (Ab)	+	∅	+	∅	Dc, VSD	-	PCD, CtD, NSt-LD	PC	PC Class 3
[7]	Case 15 ♂	O (Ab)	∅ (-)	+	+	+	TF, PDA	Type 1	H, PCD	PC with TF	PC Class 1
[10]	Case 21 C3, ♀	O (Ab)	∅ (+)	+	+	+	TF, RVD	Type 1	CrfD	PC	PC Class 1
[19]	Case 32 ♂	O (Ab)	+	∅	+	+	ASD, VSD, LVD	Type 1	∅	PC with LVD and O	PC Class 2
[20]	Case 33 ∅	O (Ab)	∅ (+)	∅	+	+	CHD	Type 1	∅	PC	PC Class 2
[21]	Case 34 C1, ∅	O (Ab)	+	∅	+	∅	∅	Type 1	AN, CP, NSt-LD	EC and O	PC Class 3
[26]	Case 45 C3, ♂	O (Ab)	∅ (+)	+	∅	∅	-	Type 2	∅	PC	PC Class 3 BSA Type VIII STBWC IV
[31]	Case 51 ♂	O (Ab)	∅ (+)	∅	+	∅	VSD, LVD	Type 1	∅	PC	PC Class 3
[45]	Case 65 ♂	Ab	∅ (+)	+	+	+	Dc, PS, BAV	-	∅	PC	PC Class 1 BSA Type VIII STBWC IV
[46]	Case 67 ♂	Ab	(+)	+	∅	∅	VSD, LVD, PDA	Type 2	∅	PC	PC Class 3 BSA Type VIII STBWC IV
[55]	Case 97 ♂	O (Ab)	Sc Short	∅	∅	∅	VSD, TA	+	∅	PC	PC Class 3
	Case 98 ♀	Ab	Uc Short	∅	∅	∅	∅	+	TRAPS	PC	EC
	Case 99 ♂	O (Ab)	Uc Short SUA	∅	∅	∅	∅	+	AA, IM	PC	EC
[56]	Case 108 ♀	O (Ab)	∅ (+)	+	+	+	TGA	Type 1	Ee, ABS, St-SpD, NSt-LD	PC with Crch	PC Class 1 BSA Type VII SSBWC IV
[65]	Case 112 ♂	O (Ab)	∅ (+)	∅	+	∅	TGA, VSD, HRV	Type 1	∅	PC	PC Class 3
[78]	Case 136 ♀	O (Ab)	+ SUA	+	+	∅	∅	Type 1	∅	PC with SUA	PC Class 3
[86]	Case 149 ♀	(Ab)	+ Cyst	+	+	+	ASD, VSD	Type 1	∅	EC associated with PC	PC Class 1 BSA Type VIII STBWC IV
[98]	Case 156 ∅	O (Ab)	+	∅	+	+	Mc, LVD, VSD	Type 1	∅	PC with LVD	PC Class 2

**∅**, non reported; **AA**, anal atresia; **Ab**, abdominoschisis; **ABS**, amniotic band syndrome; **AN**, anencephaly; **ASD**, atrial septal defect; **BAV**, bicuspid aortic valve; **BSA**, Body Stalk Anomalies; **BWD**, body wall defect; **CD**, cardiac defects; **Crch**, craniorachischisis; **CrfD**, craniofacial dysmorphism; **CtD**, costal defects; **CHD**, congenital heart disease; **CP**, cleft palate; **Dc**, dextrocardia; **DD**, diaphragmatic defect; **Ee**, exencephaly; **EC**, *Ectopia cordis*; **ExEC**, external *ectopia cordis*; **H**, hydrocephaly; **HRV**, hypoplastic right ventricle syndrome; **IM**, intestinal malrotation; **LVD**, left ventricular diverticulum; **Mc**, mesocardia; **NSt-LD**, non-structural limb defect; **O**, omphalocele; **OD**, other defects; **PC**, Pentalogy of Cantrell; **PCD**, pulmonary congenital defect; **PD**, pericardial defect; **PDA**, patent ductus arteriosus; **PS**, pulmonary stenosis; **RVD**, right ventricular dilatation; **Sc**, supercoiled; **SSBWC**, sternal spinal body wall complex; **STBWC**, sternal body wall complex; **StD**, sternal defect; **St-SpD**, structural spinal defect; **SUA**, single umbilical artery; **TA**, tricuspid atresia; **TF**, tetralogy of Fallot; **TGA**, transposition of the great arteries; **TRAPS**, twin reversed arterial perfusion sequence; **Uc**, uncoiled; **UCD**, umbilical cord defect; **VSD**, ventricular septal defect.

**Table 5:** Supraumbilical Thoracoabdominoschisis (SUThAb) Cases with Normal Umbilical Cord (*n* = 23).

References	Case/Gender	BWD	UCD	StD	DD	PD	CD	ExEC	OD	Author’s Diagnosis	Proposed Diagnosis
[5]	Case 14 ♀	SUThAb	∅ (-)	+	∅	∅	VSD, TGA	Type 3	∅	EC	PC Class 3
[9]	Case 18 ♀	SUThAb	-	+	+	+	ASD, PDA	Type 3	CrfD, CH, St-SpD, St-LD, St-GuD	CSq	PC Class 1
[10]	Case 19 C1, ♂	SUThAb	∅ (-)	+	+	+	VSD, ASD, DORV	Type 3	NSt-GuD	PC	PC Class 1
	Case 20 C2, ♀	SUThAb	∅	+	+	∅	DORV, ASD, VSD	-	L-SE, HT, GA, AA, St-GuD, NSt-SpD	PC	PC Class 2
[12]	Case 23 C1, ♂	SUThAb	∅ (-)	+	+	+	∅	Type 1	CyH	PC with CyH	PC Class 3
[15]	Case 27 ♀	SUThAb	-	+	+	∅	∅	Type 3	AOP(R), MOP(L), BCL, PCD, St-GuD, St-LD	Midline ThAb and LD	PC Class 3
[22]	Case 39 ∅	O (SUThAb)	∅ (-)	+	∅	∅	VSD	Type 3	∅	PC	PC Class 3
[24]	Case 42 ♂	SUThAb	∅ (-)	+	+	∅	ASD, PDA	Type 3	GA, St-LD	PC and LD	PC Class 2
[42]	Case 55 ♀	SUThAb	-	+	+	+	VSD	Type 3	Ee	PC with Ee and LD	PC Class 1
[53]	Case 101 ♀	O + DRM (SUThAb)	∅ (-)	+	+	-	-	Type 3	AA, CrfD, NSt-LD, NSt-GuD	Goltz–Gorlin Syndrome and PC	PC Class 3
[57]	Case 109 ♂	O (SUThAb)	∅ (-)	+	+	+	VSD, PDA	Type 3	∅	PC with EC and VSD	PC Class 1
[62]	Case 111 ♂	(SUThAb)	∅ (-)	+	+	∅	∅	Type 3	AN	CS with AN	EC
[67]	Case 115 C2, ♀	SUThAb	∅ (-)	+	∅	+	DORV, TGA	Type 3	∅	PC	PC Class 2
[66]	Case 117 ♂	(SUThAb)	∅ (-)	+	+	+	DORV, TGA, PS, VSD	Type 3	∅	EC	PC Class 1
[70]	Case 118 ∅	O (SUThAb)	∅ (-)	+	∅	+	ASD, TF, APVR	Type 3	PCD, ABS	PC with EC, APVR and TF	PC Class 2
[28]	Case 135 ♀	O (SUThAb)	∅ (-)	+	+	+	VSD	Type 3	Hy	PC	PC Class 1
[82]	Case 140 C2, ♀	(SUThAb)	∅ (-)	+	+	+	∅	Type 3	∅	PC	PC Class 3
[87]	Case 145 ∅	O (SUThAb)	∅ (-)	+	+	+	PDA, LSVC to CS	Type 3	∅	PC with total EC and a major O	PC Class 1
[91]	Case 150 ∅	SUThAb	∅ (-)	+	∅	∅	VSD	Type 3	∅	Ec, NSt-LD	PC Class 3
[92]	Case 153 ♀	SUThAb	∅ (-)	+	∅	+	∅	Type 3	BCL, CP	EC, O, BCL and CP	PC Class 3
[99]	Case 160 ♀	O (SUThAb)	∅ (-)	+	∅	+	TF, APVR, LSVC to CS	Type 3	HR	PC and EC	PC Class 2
[100]	Case 161 ♀	SUThAb	∅ (-)	∅	∅	∅	TGA, ASD, VSD, PS	+	Ep, OmT	Ep and ThAbEC	EC
[103]	Case 164 C1, ♀	SUThAb	-	+	+	+	ASD, PDA	Type 3	∅	EC complicated by PC	PC Class 1

**∅**, not reported; **AA**, anal atresia; **ABS**, amniotic band syndrome; **AN**, anencephaly; **ASD**, atrial septal defect; **AOP**, anophthalmia; **APVR**, anomalous pulmonary venous return; **BCL**, bilateral cleft lip; **BWD**, body wall defect; **CD**, cardiac defects; **CH**, cerebellar hypoplasia; **CrfD**, craniofacial dysmorphism; **CP**, cleft palate; **CrfD**, craniofacial dysmorphism; **CS**, Cantrell syndrome; **CSq**, Cantrell sequence; **CyH**, cystic hygroma; **DD**, diaphragmatic defect; **DORV**, double-outlet right ventricle; **DRM**, diastasis of the abdominal recti muscles; **Ee**, exencephaly; **EC**, *Ectopia cordis*; **Ep**, epignathus; **ExEC**, external *ectopia cordis*; **GA**, gallbladder agenesis; **HR**, hypoplastic ribs; **HT**, hypertelorism; **Hy**, hydramnios; **L**, left; **LSVC to CS**, left superior vena cava draining to coronary sinus; **L-SE**, low-set ears; **MOP**, microphthalmia; **NSt-GuD**, non-structural genitourinary defects; **NSt-LD**, non-structural limb defect; **O**, omphalocele; **OD**, other defects; **OmT**, oromandibular tumor; **PC**, Pentalogy of Cantrell; **PCD**, pulmonary congenital defect; **PD**, pericardial defect; **PDA**, patent ductus arteriosus; **PS**, pulmonary stenosis; **R**, right; **St-GuD**, structural genitourinary defects; **St-SpD**, spinal defect; **St**-**LD**, structural limb defect; **StD**, sternal defect; **SUThAb**, supra-umbilical-thoraco-abdominoschisis; **TF**, tetralogy of Fallot; **TGA**, transposition of the great arteries; **ThAb**, thoracoabdominoschisis; **UCD**, umbilical cord defect; **VSD**, ventricular septal defect.

**Table 6:** Supraumbilical Abdominoschisis (SUAb) with Normal Umbilical Cord (*n* = 63).

References	Case/Gender	BWD	UCD	StD	DD	PD	CD	ExEC	OD	Author’s Diagnosis	Proposed Diagnosis
[3]	Case 2 L.A., ♂	SUAb	-	∅	+	∅	VSD	-	PCD	PC	PC Class 3
[4]	Case 5 J.L.C., ♀	O (SUAb)	∅ (-)	-	+	+	-	-	∅	PSDH with O	PC Class 3
	Case 6 N.K., ♀	O (SUAb)	∅ (-)	-	+	+	Dc	-	∅	PSDH with O	PC Class 3
	Case 7 A.J.T., ∅	O (SUAb)	∅ (-)	+	+	+	VSD	-	IM	PSDH with O	PC Class 1
	Case 8 M.A, ∅	O (SUAb)	∅ (-)	+	+	+	BvD	-	∅	PSDH with O	PC Class 1
	Case 9 B.G.H, ∅	O (SUAb)	∅ (-)	+	+	+	VSD, PS, DORV	-	∅	PSDH with O	PC Class 1
	Case 10 H.E.P, ∅	O (SUAb)	∅ (-)	+	+	+	VSD, PTA type IV	-	PCD	PSDH with O	PC Class 1
	Case 11 A.M.S, ∅	O (SUAb)	∅ (-)	+	+	+	VSD, ASD, PAA	-	IM	PSDH with O	PC Class 1
	Case 12 B.G.H, ∅	O (SUAb)	∅ (-)	-	+	+	HLV, APVR	-	PCD, MD, CtD, St-GuD	PSDH with O	PC Class 2
	Case 13 R.M., ∅	O (SUAb)	∅ (-)	+	+	+	-	Type 1	PCD, IM, St-LD	PSDH with O	PC Class 3
[8]	Case 16 ♀	O + DRM (SUAb)	∅ (-)	+	+	+	VSD, BvD	Type 1	CtD, SS	CS with BvD, VSD and EC	PC Class 1
[13]	Case 24 C1, ♀	O (SUAb)	∅ (-)	+	+	+	TF	-	HCy	PC	PC Class 1
	Case 25 C2, ♂	O (SUAb)	∅ (-)	+	+	+	CHD	-	NSt-GuD	PC	PC Class 1
[11]	Case 28 ♂	SUAb	∅ (-)	+	+	∅	Dc, VSD, ASD, LVD, RVH	-	∅	Partial PC	PC Class 2
[17]	Case 29 ♂	SUAb	∅ (-)	+	+	+	Mc, TF, VD, SCA	-	∅	PC	PC Class 1
[16]	Case 30 ♂	O (SUAb)	∅ (-)	+	-	-	Dc, CM, SV, PAA, TA	-	∅	PC with an intact diaphragm and pericardium	PC Class 3
[18]	Case 31 ♀	DRM, SUAb	∅ (-)	+	+	+	Dc, VSD, LVD, TF, ASD	-	∅	LVD with PC and TF	PC Class 1
[23]	Case 40 ♂	O (SUAb)	∅ (-)	+	+	+	HRV, VSD, ASD, PS	-	PCD, GA, Ps	PC with TF, GA and PS	PC Class 1
[25]	Case 41 ∅	SUAb	-	+	+	+	LVD, LSCV to CS	Type 3	∅	PC with LVD	PC Class 1
[29]	Case 46 ♀	O (SUAb)	∅ (-)	+	+	+	HLHS	Type 3	∅	PC with HLHS	PC Class 1
[27]	Case 47 ♀	SUAb	∅ (-)	+	+	+	∅	Type 3	∅	PC	PC Class 2
[28]	Case 48 ∅	O (SUAb)	∅ (-)	+	+	+	VSD	Type 3	Hy	PC	PC Class 1
[32]	Case 49 ♂	SUAb	-	+	+	+	Dc, LVA, ASD	-	NSt-GuD	PC and LVA	PC Class 1
[34]	Case 52 ∅	SUAb	∅ (-)	+	+	∅	PTA, VSD	Type 3	Ecc, Myc, H	PC with ONTD, St-SpD, NSt-LD	PC Class 2
[33]	Case 53 ♀	SUAb	∅ (-)	+	+	+	HLHS	Type 3	∅	PC with HLHS	PC Class 1
[39]	Case 56 ♂	SUAb	∅ (-)	+	+	∅	Dc, PDA, AVC, VSD, BvH, RVD	Type 3	CrfD, L-SE, CH, St-SpD, NSt-LD, NSt-GuD	Incomplete PC	PC Class 2
[35]	Case 57 ♂	SUAb	-	+	+	∅	Dc, VSD, LVD	Type 3	∅	PC	PC Class 2
37]	Case 59 ♀	SUAb	∅ (-)	+	∅	∅	TA	Type 3	H	PC with severe EC	PC Class 3
[40]	Case 60 ♀	O (SUAb)	∅ (-)	-	+	+	LVD	+	∅	PC	PC Class 2
[41]	Case 61 C1, ∅	O (SUAb)	∅ (-)	+	+	+	DORV	-	IM, NSt-GuD	PC	PC Class 1
	Case 62 C2, ∅	O (SUAb)	∅ (-)	+	+	+	ASD	-	PCD	PC	PC Class 1
	Case 63 C3, ∅	O (SUAb)	∅ (-)	+	+	+	VSD, ASD	-	PCD	PC	PC Class 1
[52]	Case 70 C3, ∅	O (SUAb)	∅ (-)	+	+	+	LVD, VSD	Type 3	∅	PC with EC	PC Class 1
	Case 71 C4, ∅	O (SUAb)	∅ (-)	+	+	+	DORV, PTA, PS, PDA, SCA	Type 3	∅	PC with EC	PC Class 1
	Case 73 C6, ∅	O (SUAb)	∅ (-)	+	+	+	SA, SV, AVC, TGA, PS, PDA	Type 3	∅	PC with EC	PC Class 1
	Case 76 C9, ∅	O (SUAb)	∅ (-)	+	+	+	DORV, PS	Type 3	∅	PC with EC	PC Class 1
	Case 78 C11, ∅	O (SUAb)	∅ (-)	+	+	+	SV, PTA	Type 3	∅	PC with EC	PC Class 1
	Case 80 C13, ∅	O (SUAb)	∅ (-)	+	+	+	DORV	Type 3	∅	PC with EC	PC Class 1
	Case 81 C14, ∅	O (SUAb)	∅ (-)	+	+	+	BVD, TF	-	∅	PC without EC	PC Class 1
	Case 84 C16, ∅	O (SUAb)	∅ (-)	+	+	+	DILV, PS	-	∅	PC without EC	PC Class 1
	Case 85 C17, ∅	O (SUAb)	∅ (-)	+	+	+	DILV, ASD, PS, PDA	-	∅	PC without EC	PC Class 1
	Case 86 C18, ∅	O (SUAb)	∅ (-)	+	+	+	VSD, ASD, PDA	-	∅	PC without EC	PC Class 1
	Case 87 C20, ∅	O (SUAb)	∅ (-)	+	+	+	LVD, DORV, PS	-	∅	PC without EC	PC Class 1
	Case 88 C21, ∅	O (SUAb)	∅ (-)	+	+	+	VSD	-	∅	PC without EC	PC Class 1
[48]	Case 90 ♀	O (SUAb)	∅ (-)	∅	+	+	ASD, VSD, PDA	-	∅	PC	PC Class 2
[55]	Case 100 ♂	O (SUAb)	-	+	+	+	Dc, APVR	Type 3	∅	PC	PC Class 1
[54]	Case 102 ♂	O (SUAb)	∅ (-)	+	+		AVC, ASD, VSD, TGA, PS	Type 3	∅	PC with complex cardiac malformations	PC Class 2
[58]	Case 103 ♂	O (SUAb)	-	∅	∅	∅	∅	+	St-SpD, St-LD	PC	EC
[59]	Case 107 C3, ♂	O (SUAb)	∅ (-)	+	+	+	ASD	-	∅	PC	PC Class 1
[72]	Case 121 ♀	O (SUAb)	∅ (-)	∅	+	+	LVD, DORV, VSD, PAH, TGA, HRV	+	∅	PC with EC	PC Class 2
[73]	Case 130 ♀	SUAb	∅ (-)	+	+	∅	VSD, ASD	Type 3	∅	PC Class 2	PC Class 2
[75]	Case 132 ∅	O (SUAb)	∅ (-)	∅	+	∅	∅	+	CyH, St-SpD	PC	EC
[76]	Case 133 ♂	SUAb	∅ (-)	+	+	+	VSD	Type 3	HD, NSt-GuD	PC with unilateral kidney evisceration	PC Class 1
[77]	Case 134 ♀	O (SUAb)	∅ (-)	+	+	+	VSD, ASD, LVD	Type 3	∅	PC	PC Class 1
[82]	Case 139 C1, ♀	O (SUAb)	∅ (-)	∅	+	+	VSD, TF	Type 3	NSt-LD	PC	PC Class 2
[79]	Case 141 ♀	O + DRM (SUAb)	∅ (-)	+	+	∅	VSD, ASD, APVR, PDA, LVD	Type 3	∅	PC	PC Class 2
[84]	Case 142 ∅	SUAb	∅ (-)	∅	+	+	∅	+	UAOP, CrfD	PC with UAOP	PC Class 3
[88]	Case 147 C1, ♀	O (SUAb)	-	+	+	+	VSD, PDA, PS	Type 3	∅	PC	PC Class 1
[90]	Case 151 ♂	O (SUAb)	∅ (-)	+	+	+	ASD, VSD	Type 3	∅	PC	PC Class 1
[94]	Case 152 ♂	O (SUAb)	∅ (-)	+	+	∅	CHD	Type 3	SpD	PC with EC	PC Class 2
[97]	Case 159 C2, ∅	O (SUAb)	∅ (-)	∅	+	∅	∅	+	∅	PC	EC
[101]	Case 162 ♂	O (SUAb)	∅ (-)	+	+	∅	∅	Type 3	AH, St-SpD, NSt-LD	Complete PC with EC and multiple anomalies	PC Class 3
[102]	Case 163 ♂	O + DRM (SUAb)	∅ (-)	+	+	∅	TF	Type 3	∅	PC with TF and Absent Diaphragm	PC Class 2

**∅**, not reported; **AH**, alobar holoprosencephaly; **ASD**, atrial septal defect; **APVR**, anomalous pulmonary venous return; **AVC**, atrioventricular canal; **BvH**, biventricular hypertrophy; **BvD**, biventricular diverticulum; **BWD**, body wall defect; **CD**, cardiac defects; **CH**, cerebellar hypoplasia; **CHD**, congenital heart disease; **CrfD**, craniofacial dysmorphism; **CtD**, costal defects; **CM**, cardiomegaly; **CrfD**, craniofacial dysmorphism; **CS**, Cantrell syndrome; **CyH**, cystic hygroma; **Dc**, dextrocardia; **DD**, diaphragmatic defect; **DILV**, double-inlet left ventricle; **DORV**, double-outlet right ventricle; **DRM**, diastasis of the abdominal recti muscles; **EC**, *Ectopia cordis*; **Ecc**, encephalocele; **ExEC**, external *ectopia cordis*; **GA**, gallbladder agenesis; **H**, hydrocephaly; **HD**, hepatic defect; **HCy**, hepatic cyst; **HLHS**, hypoplastic left heart syndrome; **HLV**, hypoplastic left ventricle; **HRV**, hypoplastic right ventricle syndrome; **Hy**, hydramnios; **IM**, intestinal malrotation; **L-SE**, low-set ears; **LVA**, left ventricular aneurysm; **LVD**, left ventricular diverticulum; **Mc**, mesocardia; **MD**, musculoskeletical deformities; **Myc**, myelomeningocele; **NSt**-**GuD**, genitourinary defects; **NSt-LD**, non-structural limb defect; **O**, omphalocele; **OD**, other defects; **ONTD**, open neural tube defect; **PAA**, pulmonary artery atresia; **PAH**, pulmonary artery hipoplasia; **PC**, Pentalogy of Cantrell; **PCD**, pulmonary congenital defect; **PD**, pericardial defect; **PDA**, patent ductus arteriosus; **PTA**, persistent truncus arteriosus; **Ps**, polisplenia; **PS**, pulmonary stenosis; **PSDH**, pars sternalis diaphrgmatic hernia; **PTA**, persistent truncus arteriosus; **RVD**, right ventricular dilatation; **RVH**, right ventricular hipertrofy; **SA**, single atrium; **SCA**, single coronary artery; **SpD**, spinal defect; **SS**, *situs solitus*; **St-LD**, structural limb defect; **St-GuD**, structural genitourinary defects; **St-SpD**, spinal defect; **StD**, sternal defect; **SUAb**, supra-umbilical-abdominoschisis; **SV**, single ventricle; **TA**, tricuspid atresia; **TF**, tetralogy of Fallot; **TGA**, transposition of the great arteries; **UAOP**, unilateral anophthalmia; **UCD**, umbilical cord defect; **VD**, ventricular diverticulum; **VSD**, ventricular septal defect.

**Table 7:** Supraumbilical Incomplete Central Defect (SUICD) (*n* = 28).

References	Case/Gender	BWD	UCD	StD	DD	PD	CD	ExEC	OD	Author’s Diagnosis	Proposed Diagnosis
[10]	Case 22 C4, ♀	VEH (SUICD)	-	+	∅	+	VSD, ASD, AVS, PDA	-	Hy, L-SE, CP, St-GuD, NSt-SpD	PC	PC Class 2
[30]	Case 50 ∅	(SUICD)	-	+	∅	∅	VSD, ASD, URC, LVD	Type 3	∅	PC	PC Class 3
[43]	Case 64 ♀	(SUICD)	∅ (-)	+	+	+	PTA, VSD, ASD, PDA	Type 3	∅	PC with ThAbEC	PC Class 1
[44]	Case 66 ♀	(SUICD)	-	∅	+	+	VSD, ASD	+	∅	PC	PC Class 2
[52]	Case 72 C5, ∅	RD (SUICD)	∅ (-)	+	+	+	CoA, VSD	Type 3	∅	PC with EC	PC Class 1
	Case 74 C7, ∅	RD (SUICD)	∅ (-)	+	+	+	SV, LSVC to CS	Type 3	∅	PC with EC	PC Class 1
	Case 75 C8, ∅	RD (SUICD)	∅ (-)	+	+	+	LVD, ASD	Type 3	∅	PC with EC	PC Class 1
	Case 77 C10, ∅	RD (SUICD)	∅ (-)	+	+	+	DORV, SCA	Type 3	∅	PC with EC	PC Class 1
	Case 79 C12, ∅	RD (SUICD)	∅ (-)	+	+	+	LVD, VSD	Type 3	∅	PC with EC	PC Class 1
	Case 83 C15, ∅	RD (SUICD)	∅ (-)	+	+	+	HRVS, PTA, VSD, LSVC to CS	-	∅	PC without EC	PC Class 1
[49]	Case 92 ♀	RD (SUICD)	∅ (-)	+	+	+	Mc, DORV, VSD, LVD	Type 3	∅	PC	PC Class 1
[47]	Case 93 C1, ♀	(SUICD)	-	+	∅	∅	∅	Type 3	∅	PC	PC Class 3
	Case 94 C2, ♀	(SUICD)	-	+	∅	∅	Mc, GH	Type 3	∅	PC	PC Class 3
	Case 95 C3, ♂	(SUICD)	-	+	∅	∅	Mc, GH	Type 3	∅	PC	PC Class 3
[59]	Case 106 C2, ♀	RD (SUICD)	∅ (-)	+	+		DORV, PAA, VSD, PDA	Type 3	∅		PC Class 2
[63]	Case 116 ♂	(SUICD)		+	+	+	∅	Type 3	HD	Incomplete PC	PC Class 3
[71]	Case 122 C1, ♀	(SUICD)	∅ (-)	+	+	∅	DORV, VSD, LSVC	Type 3	∅	PC	PC Class 2
	Case 123 C2, ♂	(SUICD)	∅ (-)	+	+	∅	VSD, LSVC	Type 3	CL	PC	PC Class 2
	Case 124 C3, ♀	(SUICD)	∅ (-)	+	+	∅	ASD	-	∅	PC	PC Class 2
	Case 125 C4, ♂	(SUICD)	∅ (-)	+	+	∅	VSD	Type 3	∅	PC	PC Class 2
	Case 126 C5, ♂	(SUICD)	∅ (-)	+	+	∅	DORV, VSD, ASD, PS, LSVC	Type 3	∅	PC	PC Class 2
	Case 127 C6, ♂	(SUICD)	∅ (-)	+	+	∅	VSD, ASD, LSVC	-	PCD	PC	PC Class 2
	Case 128 C7, ♂	(SUICD)	∅ (-)	+	+	∅	VSD, ASD, LSVC	-	∅	PC	PC Class 2
	Case 129 C8, ♂	(SUICD)	∅ (-)	+	+	∅	VSD, ASD, LVD, LSVC	Type 3	CL	PC	PC Class 2
[81]	Case 138 ♂	(SUICD)	-	+	+	+	HLV	Type 3	CrfD, ABS	PC with severe amputations	PC Class 1
[83]	Case 144 ♂	RD (SUICD)	∅ (-)	∅	∅	∅	TA, VDS, PAH	+	∅	PC	EC
[95]	Case 155 ♂	RD (SUICD)	∅ (-)	+	+	+	LVD, ASD, VSD, PDA	+	∅	PC	PC Class 1
[96]	Case 157 ♂	RD (SUICD)	-	+	-	+	VSD, ASD, CoA, PDA, PLSVC	Type 3	∅	PC	PC Class 2

**∅**, not reported; **ABS**, amniotic band syndrome; **ASD**, atrial septal defect; **AVS**, aortic valve stenosis; **BWD**, body wall defect; **CD**, cardiac defects; **CrfD**, craniofacial dysmorphism; **CL**, cleft lip; **CoA**, coarctation of the aorta; **CP**, cleft palate; **CrfD**, craniofacial dysmorphism; **DD**, diaphragmatic defect; **DORV**, double-outlet right ventricle; **EC**, *Ectopia cordis*; **ExEC**, external *ectopia cordis*; **GH**, globular heart; **HD**, hepatic defect; **HLV**, hypoplastic left ventricle; **HRVS**, hypoplastic right ventricle syndrome; **Hy**, hydramnios; **LSVC to CS**, left superior vena cava draining to coronary sinus; **L-SE**, low-set ears; **LVD**, left ventricular diverticulum; **Mc**, mesocardia; **NSt-SpD**, non-structural spinal defect; **OD**, other defects; **PAA**, pulmonary artery atresia; **PAH**, pulmonary artery hipoplasia; **PC**, Pentalogy of Cantrell; **PCD**, pulmonary congenital defect; **PD**, pericardial defect; **PDA**, patent ductus arteriosus; **PLSVC**, persistent left superior vena cava; **PS**, pulmonary stenosis; **PTA**, persistent truncus arteriosus; **RD**, rectal diastasis; **SCA**, single coronary artery; **St-GuD**, genitourinary defects; **StD**, sternal defect; **SUICD**, supra-umbilical central defect; **SV**, single ventricle; **TA**, tricuspid atresia; **UCD**, umbilical cord defect; **URC**, unroofed coronary sinus; **VEH**, ventral epigastric hernia; **VSD**, ventricular septal defect.

**Table 8:** Umbilical Hernia (*n* = 10).

References	Case/Gender	BWD	UCD	StD	DD	PD	CD	ExEC	OD	Author’s Diagnosis	Proposed Diagnosis
[14]	Case 26 ♂	UH, RD	∅ (-)	+	+	+	VSD, ASD, TA, PS	Type 3	∅	PC	PC Class 1
[38]	Case 58 ♂	UH	∅ (-)	∅	+	∅	LVD, DORV	+	∅	PC with DORV	PC Class 3
[52]	Case 68 C1, ∅	UH	∅ (-)	+	+	+	SA, SV, AvC, PS	Type 3	∅	PC with EC	PC Class 1
	Case 69 C2, ∅	UH	∅ (-)	+	+	+	LVD, VSD, PDA, SCA	Type 3	∅	PC with EC	PC Class 1
	Case 82 ∅	UH	∅ (-)	+	+	+	LVD, DORV	-	∅	PC without EC	PC Class 1
	Case 89 C22, ∅	UH	∅ (-)	+	+	+	LVD, DORV	-	∅	PC without EC	PC Class 1
[50]	Case 91 ♀	UH RD	∅ (-)	∅	∅	∅	SS, ASD, LVD	+	∅	PC	PC Class 3
[60]	Case 104 ♀	UH	∅ (-)	+	+	+	Dc, LVD, triatrial SS VSD, ASD, PDA	Type 3	∅	LVD with partial PC	PC Class 1
[67]	Case 114 C1, ♀	UH, RD	∅ (-)	+	+	∅	Dc, DORV, TGA, LSVC to CS	Type 3	SS	PC	PC Class 2
[88]	Case 148 C2, ♂	UH	-	+	∅	∅	ASD, LSVC to CS	Type 3	∅	Incomplete PC	PC Class 3

**∅**, not reported; **ASD**, atrial septal defect; **AvC**, atrioventricular canal; **BWD**, body wall defect; **CD**, cardiac defects; **Dc**, dextrocardia; **DD**, diaphragmatic defect; **DORV**, double-outlet right ventricle; **EC**, *Ectopia cordis*; **ExEC**, external *ectopia cordis*; **LSVC to CS**, left superior vena cava draining to coronary sinus; **LVD**, left ventricular diverticulum; **OD**, other defects; **PC**, Pentalogy of Cantrell; **PD**, pericardial defect; **PDA**, patent ductus arteriosus; **PS**, pulmonary stenosis; **RD**, rectal diastasis; **SA**, single atrium; **SCA**, single coronary artery; **SS**, *situs solitus*; **StD**, sternal defect; **SV**, single ventricle; **TA**, tricuspid atresia; **TGA**, transposition of the great arteries; **UCD**, umbilical cord defect; **UH**, umbilical hernia; **VSD**, ventricular septal defect.

**Table 9:** Lateral Abdominal Wall Defects (*n* = 9).

References	Case/Gender	BWD	UCD	StD	DD	PD	CD	ExEC	OD	Author’s Diagnosis	Proposed Diagnosis
[21]	Case 35 C2, ∅	ThG	-	+	∅	∅	∅	Type 4	∅	EC and ThG	PC Class 3
	Case 36 C3, ∅	ThG	-	+	∅	∅	∅	Type 4	∅	EC and ThG	PC Class 3
[22]	Case 38 C1, ♀	LThAb	(-)	+	+	+	VSD	Type 1	St-SpD, NSt-LD	PC	PC Class 1
[26]	Case 43 C1, ♀	O (LThAb)	∅ (-)	+	∅	∅	-	Type 3	Ee, Hy, St-SpD, NSt-LD	PC	PC Class 3
	Case 44 C2, ♀	O (LAb/G)	∅ (-)	∅	∅	∅	-	+	Ee, Hy, St-SpD, NSt-LD	PC	EC
[36]	Case 54 ♀	ThAb (LAb/G)	-	∅	∅	∅	∅	+	Ht, CrfD, NSt-LD	EC	EC
[59]	Case 105 C1, ♂	O + RD (LAb/G)	∅ (-)	+	+	+	∅	Type 4	∅	PC	PC Class 3
[61]	Case 110 ♂	O (LThAb)	(-)	+	+	+	ASD, VSD, PDA	Type 1	BCL	PC	PC Class 1
[74]	Case 131 ♂	O (LAb/G)	∅ (-)	+	+	∅	VSD	-	∅	PC	PC Class 2

**∅**, not reported; **ASD**, atrial septal defect **BCL**, cleft lip; **BWD**, body wall defect; **CD**, cardiac defects; **CrfD**, craniofacial dysmorphism; **DD**, diaphragmatic defect; **Ee**, exencephaly; **EC**, *Ectopia cordis*; **ExEC**, external *ectopia cordis*; **G**, gastrochisis; **Ht**, hypertelorism; **Hy**, hydramnios; **LAb**, lateral abdominoschisis; **LThAb**, lateral thoracoabdominoschisis; **NSt-LD**, non-structural limb defect; **O**, omphalocele; **OD**, other defects; **PC**, Pentalogy of Cantrell; **PD**, pericardial defect; **PDA**, patent ductus arteriosus; **RD**, rectal diastasis; **StD**, sternal defect; **St-SpD**, structural spinal defect; **ThAb**, thoracoabdominoschisis; **ThG**, thoracogastroschisis; **UCD**, umbilical cord defect; **VSD**, ventricular septal defect.

**Table 10:** Special Cases (*n* = 3).

References	Case/Gender	BWD	UCD	StD	DD	PD	CD	ExEC	OD	Author’s Diagnosis	Proposed Diagnosis
[51]	Case 96 ♂	-	-	∅	+	+	ASD, APVR	-	∅	Incomplete PC	CHD
[85]	Case 143 ♀	-	-	+	∅	∅	VSD, SCA, ASD	+	Ecc, CrfD, CP	PC	EC
[103]	Case 165 C2, ♀	UICD	+	+	+	+	ASD, TF, PDA	Type 2	∅	EC complicated by PC	PC Class 1 BSA Type VIII STBWC IV

**∅**, not reported; **APVR**, anomalous pulmonary venous return; **ASD**, atrial septal defect; **BWD**, body wall defect; **CD**, cardiac defects; **CHD**, congenital heart disease; **CP**, cleft palate; **CrfD**, craniofacial dysmorphism; **DD**, diaphragmatic defect; **EC**, *ectopia cordis*; **Ecc**, encephalocele **ExEC**, external *ectopia cordis*; **OD**, other defects; **PC**, Pentalogy of Cantrell; **PD**, pericardial defect; **SCA**, single coronary artery; **UCD**, umbilical cord defect; **UICD**, umbilical incomplete central defect; **VSD**, ventricular septal defect.

**Table 11:** Summary of Literature Reviewed: Carnivore Cases Classification and Proposed Diagnosis Following Critical Data Analysis.

References	Case/ Species/ Gender	BWD	UCD	StD	DD	PD	CD	ExEC	OD	Author’s Diagnosis	Proposed Diagnosis
[127]	Case 1 D, ♂	UH	∅ (-)	∅	+	∅	∅	+	∅	CDH an UH	PC Class 3
[128]	Case 2 D, C1, ♀	(SUICD)	∅ (-)	+	+	+	VSD	Type 3	∅	CAWD, StD, DD, PD and CD	PC Class1
	Case 3 D, C2, ♀	(SUICD)	∅ (-)	+	+	+	VSD	Type 3	∅	CAWD, StD, DD, PD and CD	PC Class1
	Case 4 D, C3, ♂	(SUICD)	∅ (-)	+	+	+	VSD	Type 3	∅	CAWD, StD, DD, PD and CD	PC Class1
	Case 5 D, C4, ♂	(SUICD)	∅ (-)	+	+	+	-	Type 3	∅	CAWD, StD, DD and PD	PC Class 3
	Case 6 D, C5, ♂	(SUICD)	∅ (-)	+	+	+	-	Type 3	∅	CAWD, StD, DD and PD	PC Class 3
[129]	Case 7 D, ♂	UH	∅ (-)	+	+	+	PDA, PLCVC	Type 3	∅	Sternal cleft associated with PC	PC Class1
[130]	Case 8 D, ♂	UH	∅ (-)	+	+	+	∅	-	∅	PPDH	PC Class 3
[131]	Case 9 D, ♂	DRM (SUICD)	∅ (-)	+	+	+	∅	Type 3	∅	Incomplete PC	PC Class 3
[132]	Case 10 D, ♂	UH	∅ (-)	+	+	+	∅	-	Pericardial pseudocyst	Unusual PPDH associated with a pericardial pseudocyst	PC Class 3
[108]	Case 11 D, C1, ♂	ThAb	∅ (+)	+	+	+	NS	Type 1	St-LD,	PC	PC Class 3 BSA TYPE V STLBWC III
	Case 12 D, C2, ♂	Ab	∅ (+)	+	+	∅	NS	-	∅	PC	PC Class 3 BSA TYPE VIII STBWC IV
[133]	Case 13 D, ♂	ThAb	∅ (+)	+	-	-	-	Type 2	∅	Thoracic EC, sternal agenesis, partial ectopia hepática and fissure abdominalis	BSA TYPE VI STBWC III
[104]	Case 14 D, ♀	ThAb	+	+	+	+	MVS, ASD, HLV, TVD	Type 1	BCh, PP, ABS	PC Class 1 BSA TYPE VI STBWC III ABS	PC Class 1 BSA TYPE VI STBWC III
	Case 15 D, ♂	ThAb	+	+	+	+	GH, VSD, RVH	Type 1	SP, St-SpD, NSt-GuD	BSA TYPE V SSBWC III PC Class 1	PC Class 1 BSA TYPE V SSBWC III
	Case 16 D, ♂	LThAb	-	+	+	-	RVH	Type 4	NSt-SpD, NSt-GuD	PC Class 2	PC Class 2
[134]	Case 17 Ct, ♂	-	∅ (-)	∅	+	+	Dc	+	HF	Cardiac malposition (EC)	EC
[135]	Case 18 Ct, ♀	UH	∅ (-)	+	+	+	∅	Type 3	∅	StD with PC	PC Class 3
[136]	Case 19 Ct, ♀	(SUICD)	∅ (-)	+	+	+	AVS, BAV, DA	Type 3	Ect, SL, BG, IDBK	PC with Ect	PC Class 1

**∅**, not reported; **Ab**, abdominoschisis; **ABS**, amniotic band syndrome; **ASD**, atrial septal defect; **AVS**, aortic valve stenosis; **BAV**, bicuspid aortic valve; **BCh**, bilateral cheiloschisis; **BG**, bilobed gallbladder; **BSA**, body stalk anomaly; **BWD**, body wall defect; **CDH**, congenital diaphragmatic hernia; **CAWD**, cranioventral abdominal wall defect; **CD**, cardiac defects; **Ct**, cat; **GH**, globular heart; **D**, dog; **DA**, dextroposition of the aorta; **Dc**, dextrocardia; **DD**, diaphragmatic defect; **DRM**, diastasis of the abdominal recti muscles; **Ect**, ectrodactyly; **ExEC**, external *ectopia cordis*; **HF**, hepatic fibrosis; **HLV**, hypoplasia of the left ventricle; **IDBK**, increased distance between the kidneys and the adrenal glands; **LThAb**, lateral thoracoabdominoschisis; **MVS**, mitral valve stenosis; **NS**, non studied; **NSt-GuD**, non structural genitourinary defects; **NSt-SpD**, non structural spinal defect **OD**, other defects; **PD**, pericardial defect; **PDA**, patent ductus arteriosus; **PLCVC**, persistent left cranial vena cava; **PP**, primary palatoschisis; **PPDH**, peritoneo-pericardial diaphragmatic hernia; **RVH**, right ventricular hypertrophy; **SL**, Split liver; **SP**, secondary palatoschisis; **SSBWC**, spinal sternal body wall complex; **STBWC**, sternal body wall complex; **StD**, sternal defect; **St-LD**, structural limb defect; **St-SpD**, structural spinal defect; **SUICD**, supraumbilical incomplete central defect; **ThAb**, thoracoabdominoschisis; **TVD**, tricuspid valve dysplasia; **UCD**, umbilical cord defect; **UH**, umbilical hernia; **VSD**, ventricular septal defect.

**Table 12:** Summary of Literature Reviewed: Porcine Cases Classification and Proposed Diagnosis Following Critical Data Analysis.

References	Case/ Species/ Gender	BWD	UCD	StD	DD	PD	CD	ExEC	OD	Author’s Diagnosis	Proposed Diagnosis
[105]	Case 20 C1, ♂	(ThAb)	+ Short ACP DUV HUA	+	+	+	ASD	Type 1	EcC	Cantrell Syndrome	PC Class 1 BSA Type VI STBWC III
	Case 21 C2, ♀	(ThAb)	+ Short ACP DUV	+	+	+	VSD, GH	Type 1	EcL	Cantrell Syndrome	PC Class 1 BSA Type VI STBWC III
	Case 22 C3, ♂	(ThAb)	+ Short ACP DUV HUA	+	+	+	VSD, GH	Type 1	∅	Cantrell Syndrome	PC Class 1 BSA Type VI STBWC III
	Case 23 C4, ♂	(ThAb)	+ Short ACP DUV SUA	+	+	+	ASD, AMV, SCA	Type 1	LAM	Cantrell Syndrome	PC Class 1 BSA Type VI STBWC III
	Case 24 C5, ♀	(ThAb)	+ Short ACP DUV	+	+	+	HAs, TGA, VSD	Type 1	∅	Cantrell Syndrome	PC Class 1 BSA Type VI STBWC III
	Case 25 C6, ♀	(ThAb)	+ Short ACP DUV	+	+	+	ASD	Type 1	∅	Cantrell Syndrome	PC Class 1 BSA Type VI STBWC III

**∅**, not reported; **ACP**, abnormal coiling pattern; **AMV**, atresia of the mitral valve; **ASD**, atrial septal defect; **BSA**, body stalk anomaly; **BWD**, body wall defect; **CD**, cardiac defects; **GH**, globular heart; **DD**, diaphragmatic defect; **DUV**, dispersed umbilical vessels; **EcC**, ectopic caecum; **EcL**, ectopic liver; **ExEC**, external *ectopia cordis*; **HAs**, hypoplastic auricles; **HUA**, hypoplastic umbilical artery; **LAM**, liver amorphous mass without lobulation; **OD**, other defects; **PD**, pericardial defect; **SCA**, single coronary artery; **STBWC**, sternal body wall complex; **StD**, sternal defect; **SUA**, single umbilical artery; **TGA**, transposition of the great arteries; **ThAb**, thoracoabdominoschisis; **UCD**, umbilical cord defect; **VSD**, ventricular septal defect.

**Table 13:** Summary of Literature Reviewed: Classification and Proposed Diagnosis of Ruminant Cases Following Critical Data Analysis.

References	Case/ Species/ Gender	BWD	UCD	StD	DD	PD	CD	ExEC	OD	Author’s Diagnosis	Proposed Diagnosis
[137]	Case 26 Cf, C1, ♀	-	∅ (-)	+	∅	(+)	DbA, CVCD	+	AADT, NSt-SpD	Cervical EC	EC
	Case 27 Cf, C2, ♀	-	∅ (-)	+	∅	(+)	DbA, CVCD	+	AADT, CP	Cervical EC	EC
	Case 28 Cf, C3, ♂	-	∅ (-)	+	∅	(+)	DbA, CVCD, VAD	+	AADT, NSt-SpD	Cervical EC	EC
	Case 29 Cf, C4, ♂	-	∅ (-)	+	∅	(+)	DbA, CVCD, PDA	+	AADT	Cervical EC	EC
	Case 30 Cf, C5, ♀	-	∅ (-)	+	∅	(+)	DbA, VAD, SCA	+	AADT, NSt-SpD	Cervical EC	EC
	Case 31 Cf, C6, ♀	-	∅ (-)	+	+	(+)	DbA, CVCD, SCA	+	AADT, NSt-SpD, CP	Cervical EC	EC
	Case 32 Cf, C7, ♂	-	∅ (-)	+	∅	(+)	DbA, CVCD, VAD	+	AADT, NSt-SpD, CSt	Cervical EC	EC
	Case 33 Cf, C8, ♂	-	∅ (-)	+	∅	(+)	DbA, CVCD	+	AADT, NSt-SpD	Cervical EC	EC
[138]	Case 34 Cf, ♂	-	∅ (-)	+	∅	∅	CVCD, SPV	+	Chromosomal aberrations	Cervical EC	EC
[137]	Case 35 Cf, C1, ♂	-	∅ (-)	+	∅	+	GH, DbA, CVCD, VAD, SPV	+	St-SpD, NSt-GuD, CP	Cervico-pectoral EC	EC
	Case 36 Cf, C2, ♀	-	∅ (-)	+	∅	+	GEH, SPV, RAV	+	NSt-SpD, NSt-GuD, CP	Cervico-pectoral EC	EC
[139]	Case 37 Cf, ♀	-	∅ (-)	+	∅	+	APVR	+	HF, LAM, FK, HHS	Total pectoral EC and other congenital malformations	EC
[140]	Case 38 Cf, ♀	UH	∅ (-)	∅	+	+	ASD, VSD, DORV, PDA	+	SIL	PC with Taussig-Bing syndrome and SIL	PC Class 2
[141]	Case 39 Cf, ♀	-	∅ (-)	+	∅	∅	CHt, DTV	+	∅	Cervical EC	EC
[142]	Case 40 Lm, C1, ♂	-	∅ (-)	+	∅	+	∅	+	∅	Complete Thoracic EC	EC
	Case 41 Lm, C2, ♂	-	∅ (-)	+	∅	+	∅	+	CtD	Complete Thoracic EC	EC

**∅**, non reported; **AADT**, aortic arch dog type; **APVR**, anomalous pulmonary venous return; **ASD**, atrial septal defect; **BWD**, body wall defect; **CD**, cardiac defects; **Cf**, calf; **CHt**, cardiac heterotaxia; **CP**, cleft palate; **CSt**, colonic stenosis; **CtD**, costal defects; **CVCD**, cranial vena cava duplicated; **DbA**, double apex; **DD**, diaphragmatic defect; **DORV**, double-outlet right ventricle; **DTV**, dysplasia of the tricuspid valve; **ExEC**, external *ectopia cordis*; **FK**, fibrotic kidney; **GEH**, grossly enlarged heart; **GH**, globular heart; **HF**, hepatic fibrosis; **HHS**, hyperplastic and hard spleen; **LAM**, liver amorphous mass without lobulation; **Lm**, Lamb; **NSt-GuD**, non-structural genitourinary defect; **NSt-SpD**, non-structural spinal defect; **OD**, other defects; **PD**, pericardial defect; **PDA**, patent ductus arteriosus; **RAV**, right azygos vein; **SCA**, single coronary artery; **SIL**, *situs inversus* of the liver; **SPV**, single pulmonary vein; **StD**, sternal defect; **St-SpD**, structural spinal defect; **UCD**, umbilical cord defect; **UH**, umbilical hernia; **VAD**, vena azygos duplicated; **VSD**, ventricular septal defect.

**Table 14:** Comparative Embryological Summary of Ventral Body Wall Defects in Humans and Dogs: Developmental Origins, Timing and Characteristic Features.

Defect	Embryologic Origin	Timing (Human)	Timing (Dog)	Characteristic Features
Omphalocele	Failure of midgut return after physiologic herniation	Weeks 6–10	~Days 30–35	Sac-covered herniation at umbilicus
Supraumbilical defect	Failure of lateral plate mesoderm fusion at ventral midline	Days 14–18	Days 14–35	Multisystem anomalies (sternum, diaphragm, pericardium, abdominal wall)
Sternal defect	Incomplete fusion of paired sternal bars (somatic mesoderm)	Days 14–18	Days 14–35	Sternal cleft or agenesis
Diaphragmatic defect	Abnormal septum transversum and pleuroperitoneal membrane incorporation	Days 14–18	Days 14–35	Ventral diaphragmatic gaps, often with Cantrell’s spectrum
Gastroschisis	Localized disruption of lateral body wall folding	Weeks 4–6	~Days 30–35	Paraumbilical opening, no sac, isolated defect
Rectus diastasis	Incomplete fusion of linea alba (lateral plate mesoderm)	Days 14–18	Days 14–35	Separation of rectus muscles, no true wall defect

## Data Availability

No new data were created or analyzed in this study. Data sharing is not applicable to this article.

## Abbreviations

The following abbreviations are used in this manuscript:

AA	Anal atresia
AAA	Aplasia of the aortic arch
AADT	Aortic arch dog type
AAH	Anterior abdominal hernia
Ab	Abdominoschisis
AbEC	Abdominal *ectopia cordis*
ABS	Amniotic band syndrome
ACP	Abnormal coiling pattern
AE	Adrenal ectopia
AH	Alobar holoprosencephaly
AMV	Atresia of the mitral valve
AN	Anencephaly
ASD	Atrial septal defect
AOP	Anophthalmia
APVR	Anomalous pulmonary venous return
AvC	Atrioventricular canal
AVS	Aortic valve stenosis
BAV	Bicuspid aortic valve
BCh	Bilateral cheiloschisis
BG	Bilobed gallbladder
BvD	Biventricular diverticulum
BvH	Biventricular hypertrophy
BWD	Body wall defect
CA	Cerebellar aplasia
CAWD	Cranioventral abdominal wall defect
Cch	Cranioschisis
CD	Cardiac defect
CDH	Congenital diaphragmatic hernia
Cf	Calf
CH	Cerebellar hypoplasia
CHD	Congenital heart disease
CHt	Cardiac heterotaxia
CL	Cleft lip
CM	Cardiomegaly
CoA	Coarctation of the aorta
CP	Cleft palate
Crch	Craniorachischisis
CrfD	Craniofacial dysmorphism
CS	Cantrell syndrome
CSq	Cantrell sequence
CSt	Colonic stenosis
Ct	Cat
CtD	Costal defect
CVCD	Cranial vena cava duplicated
CyH	Cystic hygroma
D	Dog
DA	Dextroposition of the aorta
DbA	Double apex
Dc	Dextrocardia
DD	Diaphragmatic defect
Di	Distorted at the umbilicus
DILV	Double-inlet left ventricle
DORV	Double-outlet right ventricle
DRM	Diastasis of the abdominal recti muscles
DTV	Dysplasia of the tricuspid valve
DUV	Dispersed umbilical vessels
EC	*Ectopia cordis*
Ecc	Encephalocele
EcC	Ectopic caecum
EcL	Ectopic liver
Ect	Ectrodactyly
Ee	Exencephaly
EH	Epigastric hernia
Ep	Epignathus
ExEC	External *ectopia cordis*
FK	Fibrotic kidneys
G	Gastrochisis
GA	Gallbladder agenesis
GEH	Grossly enlarged heart
GH	Globular heart
H	Hydrocephaly
HAs	Hypoplastic auricles
HCy	Hepatic cyst
HD	Hepatic defect
HF	Hepatic fibrosis
HHS	Hyperplastic and hard spleen
HLHS	Hypoplastic left heart syndrome
HLV	Hypoplasia of the left ventricle
HRVS	Hypoplastic right ventricle syndrome
HR	Hypoplastic ribs
HT	Hypertelorism
HUA	Hypoplastic umbilical artery
Hy	Hydramnios
ICD	Intracardiac defect
IDBK	Increased distance between the kidneys and the adrenal glands
IM	Intestinal malrotation
Lm	Lamb
L	Left
LAb	Lateral abdominoschisis
LAM	Liver amorphous mass without lobulation
LSVC to CS	Left superior vena cava draining to coronary sinus
L-SE	Low-set ears
LThAb	Lateral thoracoabdominoschisis
LVA	Left ventricular aneurysm
LVD	Left ventricular diverticulum
Mc	Mesocardia
MD	Musculoskeletal deformities
MOP	Microphthalmia
MVA	Mitral valve agenesis
MVS	Mitral valve stenosis
Myc	Myelomeningocele
NSt-GuD,	Non-structural genitourinary defects
NSt-LD	Non-structural limb defect
NSt-SpD	Non-structural spinal defect
O	Omphalocele
OD	Other defects
OmT	Oromandibular tumor
ONTD	Open neural tube defect
P	Pig
PAA	Pulmonary artery atresia
PAH	Pulmonary artery hypoplasia
PCD	Pulmonary congenital defect
PD	Pericardial defect
PDA	Patent ductus arteriosus
PLSVC	Persistent left superior vena cava
PLCVC	Persistent left cranial vena cava
PP	Primary palatoschisis
PPDH	Peritoneo-pericardial diaphragmatic hernia
Ps	Polisplenia
PS	Pulmonary stenosis
PSDH	Pars sternalis diaphragmatic hernia
PTA	Persistent truncus arteriosus
R	Right
RAV	Right azygos vein
RD	Rectal diastasis
RVD	Right ventricular dilatation
RVH	Right ventricular hipertrofy
SA	Single atrium
Sc	Supercoiled
SCA	Single coronary artery
SIL	*Situs inversus* of the liver
SL	Split liver
SP	Secondary palatoschisis
SPV	Single pulmonary vein
SS	*Situs solitus*
St-GuD	Structural genitourinary defects
St-LD	Structural limb defect
St-SpD	Structural spinal defect
StD	Sternal defect
SUA	Single umbilical artery
SUAb	Supra-umbilical-abdominoschisis
SUICD	Supraumbilical incomplete central defect
SUThAb	Supra-umbilical-thoraco-abdominoschisis
SV	Single ventricle
TA	Tricuspid atresia
TF	Tetralogy of Fallot
TGA	Transposition of the great arteries
Th	Thoracoschisis
ThAb	Thoracoabdominoschisis
ThAbEC	Thoraco-abdominal *ectopia cordis*
ThG	Thoracogastroschisis
TRAPS	Twin reversed arterial perfusion sequence
TVD	Tricuspid valve dysplasia
U	Unilateral
Uc	Uncoiled
UCD	Umbilical cord defect
UH	Umbilical hernia
URC	Unroofed coronary sinus
VAD	Vena azygos duplicated
VD	Ventricular diverticulum
VEH	Ventral epigastric hernia
VH	Ventral hernia
VSD	Ventricular septal defect
